# The impact of climate and antigenic evolution on seasonal influenza virus epidemics in Australia

**DOI:** 10.1038/s41467-020-16545-6

**Published:** 2020-06-02

**Authors:** Edward K. S. Lam, Dylan H. Morris, Aeron C. Hurt, Ian G. Barr, Colin A. Russell

**Affiliations:** 1 Department of Veterinary Medicine, University of Cambridge, Cambridge, UK; 2 Department of Ecology and Evolutionary Biology, Princeton University, Princeton, NJ USA; 3 WHO Collaborating Centre for Reference and Research on Influenza, VIDRL, Peter Doherty Institute for Infection and Immunity, Melbourne, VIC Australia; 4 Department of Microbiology and Immunology, University of Melbourne, Parkville, VIC Australia; 5 School of Applied Biomedical Sciences, Federation University, Churchill, VIC Australia; 6 Department of Medical Microbiology, Academic Medical Center, University of Amsterdam, Amsterdam, The Netherlands

**Keywords:** Data integration, Population dynamics, Influenza virus

## Abstract

Although seasonal influenza viruses circulate globally, prevention and treatment occur at the level of regions, cities, and communities. At these scales, the timing, duration and magnitude of epidemics vary substantially, but the underlying causes of this variation are poorly understood. Here, based on analyses of a 15-year city-level dataset of 18,250 laboratory-confirmed and antigenically-characterised influenza virus infections from Australia, we investigate the effects of previously hypothesised environmental and virological drivers of influenza epidemics. We find that anomalous fluctuations in temperature and humidity do not predict local epidemic onset timings. We also find that virus antigenic change has no consistent effect on epidemic size. In contrast, epidemic onset time and heterosubtypic competition have substantial effects on epidemic size and composition. Our findings suggest that the relationship between influenza population immunity and epidemiology is more complex than previously supposed and that the strong influence of short-term processes may hinder long-term epidemiological forecasts.

## Abstract (web summary)

Seasonal influenza epidemics vary in timing and size, but the causes of the variation remain unclear. Here, the authors analyse a 15-year city-level data set, and find that fluctuations in climatic factors do not predict onset timing, and that while antigenic change does not have a consistent effect on epidemic size, the timing of onset and heterosubtypic competition do.

## Introduction

Seasonal influenza virus epidemics are a substantial source of disease burden and result in ~650,000 deaths each year^1^. Four co-circulating subtypes/lineages of influenza viruses currently cause disease in humans: A/H3N2 (A/H3), A/H1N1 (currently A/H1pdm09, previously A/H1seasonal (A/H1sea)), B/Victoria/2/87-like (B/Vic) and B/Yamagata/16/88-like (B/Yam) viruses. The timing, duration and size of local influenza virus epidemics can vary substantially from year to year^2,3^, but the underlying causes of this variation are poorly understood. Better understanding of the factors that govern epidemic onset and magnitude could allow for accurate and timely epidemiological forecasts^4^ and more efficient allocation of public health resources^5^.

In temperate regions of the Northern and Southern Hemispheres, influenza virus activity is most common in winter months, but the mechanistic basis of this seasonality remains unclear. Experimental studies demonstrated that reductions in temperature and absolute humidity enhance viral stability and aerosol transmission^6–8^. However, epidemics in tropical and subtropical regions often occur during periods of high temperature and humidity^9^.

Climatic fluctuations have been implicated as triggers for influenza epidemics in temperate regions. A study of state-level epidemiological data from the United States found that influenza epidemics sometimes follow 2-week periods of anomalously low absolute humidity^10^. Subsequent studies of epidemiological activity have found similar results using prefecture-level data from Japan^11^, city-level data from the New York Metropolitan Area^12^ and region-level data from France^13^.

Influenza virus evolutionary dynamics are another theorised driver of influenza virus epidemiology. Within each type and subtype of seasonal influenza virus, new major antigenic variants arise every 3–8 years^14,15^. New variants partially escape the immunity induced by prior infections and vaccinations, rendering a higher fraction of individuals susceptible to infection. Epidemiological theory predicts that epidemics caused by a new antigenic variant should therefore be larger than epidemics of previously circulated variants^16,17^.

Antigenic change could also produce earlier and more spatiotemporally synchronous epidemics. When more individuals are susceptible, fewer transmission chains go stochastically extinct, so each new introduction of a virus into a population has a higher chance of causing an epidemic. Consistent with this, studies have suggested that antigenic change is associated with earlier epidemics in Israel^18^, and with more synchronous epidemics among cities in the United States^19–21^, Japan^22^ and Australia^23^.

Studies of environmental and virological drivers of influenza virus epidemiology, including the studies referenced above, have been limited by three factors: (1) the reliance on influenza-like illness (ILI) data, (2) the aggregation of ILI or virologically confirmed data over large geographical scales (state/province/country) and (3) where virologically confirmed data are available, the use of data without subtype and antigenic variant-level resolution.

ILI data frequently include a wide variety of respiratory infections^24^, and limited laboratory characterisation obscures influenza virus type/subtype- and antigenic variant-specific patterns. These patterns become superimposed upon each other due to aggregation of ILI or virologically confirmed data to ecological scales (state/province/country) that sum over multiple local epidemics (county/city/town), which can individually vary substantially in timing, magnitude and influenza virus composition. Altogether, these sources of obfuscation make it difficult to disentangle local-level, antigenic variant-specific patterns, and critically investigate the impact of virus antigenic change.

Here, we use a 15-year data set of 18,250 typed, subtyped and antigenically characterised seasonal influenza viruses from the five most populous cities in Australia to investigate the impact of environmental and virological factors on the timing and magnitude of city-level influenza virus epidemics. We find that climatic fluctuations and virus antigenic change have no consistent effects on epidemic onset timing or size, while epidemic onset timing itself and heterosubtypic competition have substantial impacts on epidemic size and virus subtype composition. The lack of consistent effect of easily measured climatic and virus antigenic properties, and seeming dominance of noisy short-term transmission processes likely diminishes the feasibility of meaningful long-term influenza epidemic forecasting at local scales.

## Results

### Australia laboratory-confirmed influenza

**Fig. 1:**
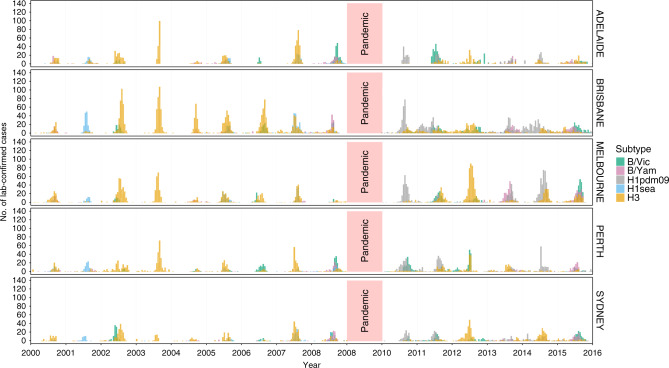
Number of laboratory-confirmed seasonal influenza virus infections from 2000 to 2015 for the five largest cities in Australia. Cases are aggregated by 2-week periods, stratified by city and coloured by subtype/lineage.

We aggregated 18,250 laboratory-confirmed and antigenically characterised cases of seasonal influenza viruses from 2000 to 2015 by 2-week (14-day) periods, creating a set of subtype- and antigenic variant-specific time series for the five most populous cities in Australia: Sydney (~5.5 million people), Melbourne (~5.0 million), Brisbane (~2.4 million people), Perth (~2.3 million) and Adelaide (~1.4 million) (Fig. 1). We excluded all virus cases from the 2009 season from all analyses because the 2009 A/H1N1 virus pandemic was atypical compared with seasonal epidemics and likely to be driven by different processes, affecting both epidemic dynamics and data collection of A/H1pdm09, as well as the other subtypes. Using a Poisson count detection method (see ‘Methods’), we identified periods of sustained, above-baseline levels of epidemic activity for each antigenic variant in each city. To facilitate comparisons among cities, we calculated the laboratory-confirmed incidence per 10^6^ individuals using the annual estimated resident population values of each city^25^.

Epidemic magnitude and the most common virus subtype varied substantially among cities (Fig. 1). For example, during the 2002 season, A/H3 and B/Vic viruses were the most common strains in both Brisbane and Sydney. Absolute A/H3 virus incidence in Brisbane was much higher than in Sydney (186 vs 38.0 cases per 10^6^ individuals), as was absolute B/Vic incidence (40.3 vs 22.7 cases per 10^6^ individuals). But B/Vic had a substantially higher relative incidence in Sydney than in Brisbane (37% of all cases, vs only 18%). In some seasons, a virus antigenic variant caused a major epidemic in one or more cities, but failed to produce any observable above-baseline activity in another city. For example, in 2006, the A/Wisconsin/67/2005 (H3N2) virus variant caused epidemics in Brisbane, Perth and Melbourne, while above-baseline levels of activity were completely absent in Adelaide.

### Effect of climatic factors

**Fig. 2:**
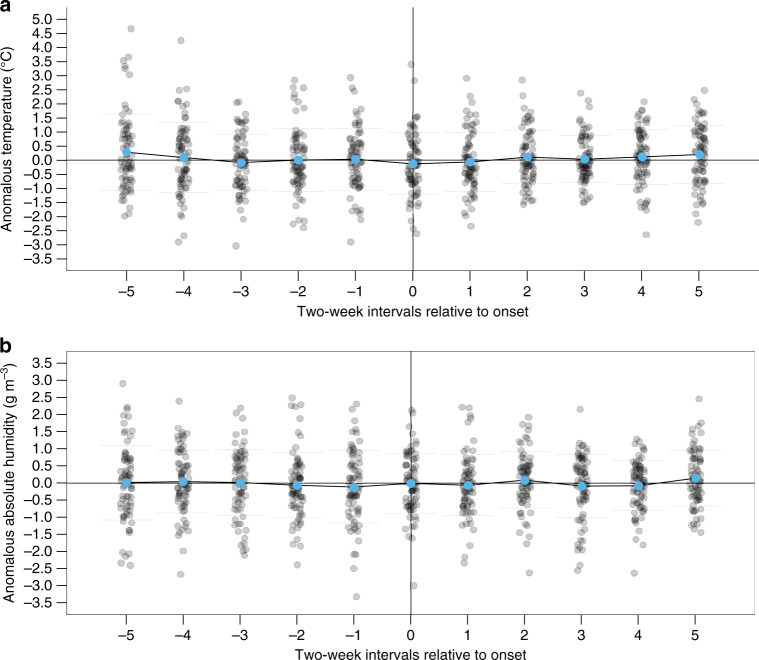
Climatic conditions around epidemic onset. **a** Anomalous temperature T′ and **b** absolute humidity AH′ prior to and after epidemic onset across all five cities. Epidemic onset is marked by the vertical line at 0. For the earliest-onset epidemic in each season and city (15 years × 5 cities = 75 epidemics), T′ and AH′ for each time point are represented by grey points: a point below the horizontal line denotes that the value is lower than the 31-year city-specific mean. Blue points show the mean T′ and AH′ for that 2-week period for all epidemics across all cities within the study period. There were no periods with statistically significant reductions (*P* < 0.05; Non-parametric bootstrapping) in T′ or AH′ from the 31-year averages.

Epidemic onset timing varied substantially within and among cities and virus subtypes (Supplementary Fig. 1). Previously, Shaman et al.^10^ showed that the 2-week period preceding the onset of state-level ILI epidemics in the United States was often marked by unusually low temperatures (T) or absolute humidities (AH). Fluctuations in these climatic factors from the historic averages expected for that specific day of the year (T′ and AH′, respectively) were anomalously large and negative when compared against a bootstrapped distribution of random samples from the historical records of observed daily climatic fluctuations recorded over wintertime (defined as 1 October–28 February). Following the same bootstrap-sampling method (see ‘Methods’) and aggregating epidemics across all five Australian cities, there were no statistically significant differences (all *P* > 0.05, see Supplementary Table 1) between the bootstrapped distribution of random samples of typical wintertime fluctuations (1 April–31 August for Australia) and the observed fluctuations in anomalous temperature and absolute humidity over the 2-, 4- and 6-week periods immediately prior to the onset of the earliest epidemics from 2000 to 2015 (excluding 2009, 15 years × 5 cities = 75 epidemics in total) (Fig. 2). Individual city-by-city analyses (Supplementary Fig. 2 and Supplementary Table 2) showed that there was substantial local variation but no consistent patterns. Epidemic onset times coincided with both high and low temperature and absolute humidity periods, and there were no statistically significant patterns in four of the five cities.

Even if anomalous fluctuations in temperature and humidity do not necessarily affect epidemic onset, climatic factors could have an impact on virus transmission^7^ and overall epidemic size: for example, influenza mortality in New York Metropolitan Area was shown to be negatively associated with temperature and humidity^12^. Overall, epidemic incidence should depend strongly on the initial exponential growth phase of the epidemic, where transmission may be facilitated by favourable climatic conditions. We therefore investigated the impact of mean temperature and mean absolute humidity during each epidemic, as well as just the period from epidemic onset to the peak, on that epidemic’s size. For both time periods considered, epidemic incidence was not associated with mean absolute humidity (Supplementary Fig. 3). We found that epidemic incidence was weakly negatively associated with the mean temperature during the epidemic and the period from start to the peak, but this relationship appears to be primarily driven by two instances, where small epidemics occurred during the early and warmer part of the season; on balance, the highly variable epidemic sizes observed over a range of climatic conditions, suggest that climatic factors have limited and noisy effects (Supplementary Fig. 3).

A recent study by Geoghegan et al.^23^ estimated epidemic onset timings for influenza A virus epidemics in Australian postcodes for the seasons from 2007 to 2016. Despite the lack of subtype-level resolution, their data set is substantially larger (450,000 entries) than the one used here, and offers an opportunity to compare findings. We repeated our anomalous temperature and absolute humidity analyses on the Geoghegan et al.^23^ data set. As with our original data set, there were no consistent statistically significant relationships between climate anomalies and epidemic onset (Supplementary Discussion, Supplementary Tables 3, 4, Supplementary Figs. 4 and 5).

Other climatic factors have been proposed as drivers of influenza dynamics, notably relative humidity and rainfall^6,9^. We repeated the above analyses for relative humidity and rainfall. There were some city-level associations, but no consistent pattern and no pattern when aggregating across cities. Epidemic onset was not associated with statistically significant fluctuations in anomalous relative humidity and rainfall.

### Effect of antigenic change

**Fig. 3:**
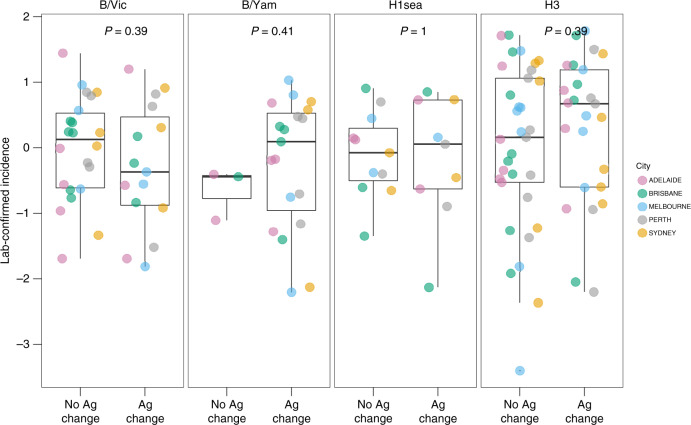
Effect of antigenic change on epidemic incidence. Epidemic incidence was compared between seasons associated with and without the epidemic-level circulation of a new major antigenic variant. Within each subtype, the incidence for individual epidemics was log-transformed and subtracted by the city-specific mean of log incidence, to allow for comparison between cities. *P* values are from Wilcoxon two-sample tests (*n* = 37, 26, 22 and 63 for B/Vic, B/Yam, A/H1sea and A/H3, respectively). Each point corresponds to one epidemic in a city, and the box plots show the median, first and third quartile of the transformed values, and range.

We next examined the effect of antigenic evolution on epidemic dynamics. Between 2000 and 2015, 7A/H3, 3A/H1sea, 1A/H1pdm09, 3 B/Vic and 5 B/Yam virus antigenic variants circulated in Australia. All A/H1pdm09 virus epidemics from 2009 to 2015 were excluded for this set of analyses for two reasons. First, we could not accurately estimate the size of the 2009 pandemic. Second, there was no subsequent, detectable antigenic change observed for A/H1pdm09 viruses during the study period. We normalised epidemic sizes (see ‘Methods') to enable comparisons between cities. Stratifying by subtype/lineage, we compared the size of the first epidemic caused by an antigenic variant against the sizes of epidemics of the same antigenic variant in subsequent years (Wilcoxon two-sample test, Fig. 3). Contrary to the predictions of previous theoretical studies^16,17^, newly emerged antigenic variants caused epidemics, both larger and smaller than city-specific mean epidemic sizes, and there was no evidence of a consistent effect of antigenic change on epidemic size.

We also compared the timing of the first epidemic caused by an antigenic variant against the timings of subsequent epidemics (Supplementary Fig. 6) to test the hypothesis that new variants cause earlier epidemics. The range of onset timings was very broad, with epidemics starting from very early to late into the season, and there were no statistically significant differences in epidemic onset timing between new and extant variant epidemics.

To investigate the impact of antigenic change on the spatiotemporal synchrony of epidemics, we examined the timing of epidemic activity across cities for years when a new major antigenic variant circulated in all five cities. New antigenic variants often failed to initiate epidemics across all five cities in a given year. We compared the synchrony of epidemics (defined as the reciprocal of the variance in epidemic onset timings) in the season in which an antigenic variant first emerges to the synchrony in subsequent seasons. There were no statistically significant differences in epidemic synchrony associated with antigenic novelty (Supplementary Fig. 7).

To check the robustness of this result, we repeated these analyses using estimated-onset timings from Geoghegan et al.^23^. There was again no discernible effect of antigenic change on the timing or synchrony of epidemics (Supplementary Discussion).

### Effect of prior immunity

**Fig. 4:**
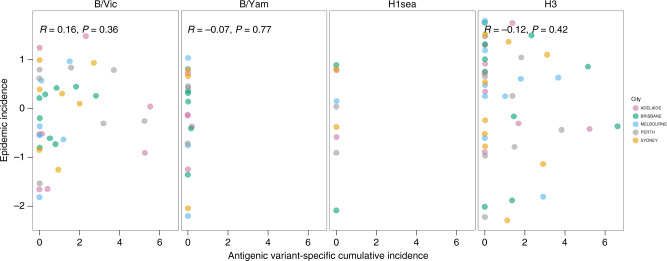
Effect of prior immunity on epidemic incidence. Within each subtype, the incidence for individual epidemics was log-transformed and subtracted by the city-specific mean of log incidence, to allow for comparison between cities. Antigenic variant-specific cumulative incidence was measured relative to the city-specific mean epidemic size, where 1 is equivalent to the mean epidemic incidence. *r*- and *P* values are from Pearson’s correlation tests (*n* = 37, 20, 9 and 45 for B/Vic, B/Yam, A/H1sea and A/H3, respectively). Antigenic variants of B/Yam rarely initiated multiple epidemics during the study period, and it was not possible to calculate a correlation coefficient for A/H1sea because the one new antigenic variant to emerge during the study period caused only a single epidemic per city.

After an antigenic variant causes an epidemic in a city for the first time, the accumulated population immunity to that variant should lead to smaller subsequent epidemics, and eventually render further epidemics of that variant less likely. For each epidemic caused by a given antigenic variant, we investigated the relationship between that epidemic’s size and the cumulative number of cases caused by that antigenic variant in preceding seasons. To account for differences in population size and surveillance intensity among cities, we normalised epidemic and cumulative case counts by the city-specific mean epidemic size. Antigenic variants that emerged prior to the start of the study period, such as A/Moscow/10/99 (A/H3) and A/New Caledonia/20/99 (A/H1sea) and all A/H1pdm09 epidemics from 2009 to 2015 were excluded from this analysis, since it was not possible to calculate cumulative case counts for them. Specific B/Yam antigenic variants rarely caused more than one epidemic in a given city, but specific antigenic variants of A/H3 and B/Vic viruses caused repeated epidemics in the same city. For A/H3 and B/Vic viruses, epidemic size and cumulative prior incidence were not correlated (Pearson’s correlation test, Fig. 4).

The accumulation of population immunity should also reduce the probability of successful epidemic initiation, making epidemics, regardless of size, less likely to start after an antigenic variant has already caused an epidemic in that city. For B/Vic and A/H1sea viruses, binary logistic regression showed non-significant associations between the cumulative incidence over prior seasons and the probability of successful epidemic initiation (all OR < 1; all *P* > 0.05, Supplementary Fig. 8 and Supplementary Table 5). This partially resulted from the small number of A/H1sea epidemics during the study period, most of which were caused by newly emerged antigenic variants. However, B/Yam and H3 viruses showed significant negative relationships between cumulative prior incidence and epidemic probability, suggesting that prior incidence may have a substantial impact on the probability of successful epidemic initiation.

### Aggregating across subtypes

There may be subtype/lineage-specific differences in the effect of antigenic change and prior immunity. Notably, B/Yam antigenic variants typically cause only one epidemic per city. We repeated these analyses with epidemics aggregated together, across all subtypes and cities to increase statistical power (see the project Github repository for the analyses and code). As before, there were no statistically significant differences in the magnitude of epidemics between the first and subsequent epidemics of an antigenic variant, or any association between epidemic size and the cumulative incidence over prior seasons. Binary logistic regression showed that the probability of successful epidemic initiation may be moderately reduced by the cumulative incidence over prior seasons. Our findings were robust to the method of normalisation used to allow for comparison between cities and subtypes/lineages (see ‘Methods').

### Effect of competition among subtypes

**Fig. 5:**
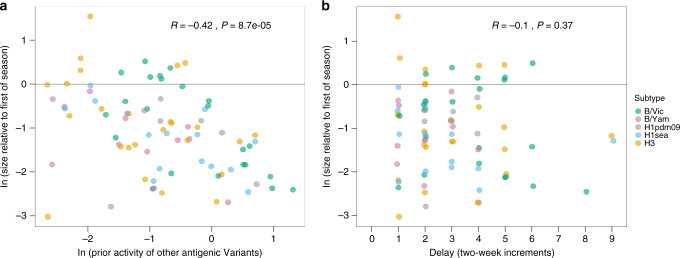
Effect of competition among subtypes on epidemic incidence. The relationship between the size of an epidemic and **a** the amount of prior activity of all other antigenic variants and subtypes, and **b** the delay in epidemic onset. The size of each epidemic, relative to the earliest epidemic of that season, was log-transformed: the horizontal line at 0 denotes that the size of an epidemic is equal to that of the earliest epidemic of that season and city. In panel (**a**), prior activity by other subtypes within the same season was measured relative to the city-specific mean epidemic size. In panel **b**, delay in epidemic onset was measured relative to the onset timing of the earliest epidemic of that season. *r*- and *P* values are from Pearson’s correlation tests (*n* = 82).

Competition among virus subtypes for hosts should create a first-mover advantage for the first subtype to sustain above-baseline epidemic activity in a city in a given season. Subsequent epidemics of other subtypes within that same season should therefore be reduced in size. We considered two proxies for this kind of intersubtypic interference: the cumulative amount of epidemic activity prior to the onset of a subtype’s epidemic and the lag between the focal epidemic and the season’s earliest epidemic. To allow for comparisons across cities and subtypes, we normalised log-epidemic case counts by subtracting off the city- and subtype-specific mean log- epidemic case count. There was a strongly negative and statistically significant correlation between prior epidemic activity and epidemic size (Pearson’s correlation test, *r* = −0.420; *P* = 8.7e–5, Fig. 5). An important caveat is that seasonality in the transmission rate could result in epidemics that start later in a season being smaller than those that started earlier, regardless of intersubtypic competition.

### Joint contributions of climatic and virological factors

Whilst the magnitude of the effects of the climatological and virological factors may be individually subtle, it could be the case that they are only able to affect observable changes on the magnitude and timing of epidemics when acting in concert, or that large effects in opposing directions mask each other. We used a Bayesian multilevel regression model to identify which putative predictor variables affected epidemic incidence, and estimate posterior distributions for their effects on epidemic size. The model included the following variables: antigenic change, cumulative prior cases of the antigenic variant, mean absolute humidity during the epidemic, activity by other subtypes earlier in the season, epidemic start date and rainfall during the epidemic. Mean temperature during the epidemic was omitted as a predictor, since it was highly collinear with absolute humidity; analyses were subsequently repeated using mean temperature, and omitting absolute humidity with no substantial changes in the overall results.

**Fig. 6:**
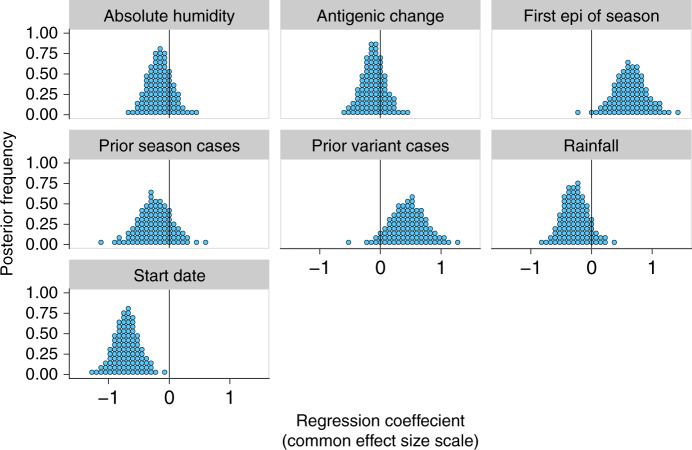
Joint contributions of climatic and virological factors on epidemic incidence. The mean effects across all subtypes were estimated using the Bayesian multilevel model. Predictors were mean-centred and scaled, so effect sizes are shown on a common scale.

The model suggested that epidemics that were the first of the season or had early start dates should be modestly larger (Fig. 6). Start date had the largest estimated effect and the clearest posterior support for a non-trivial effect size. Posterior modes for the mean effects of antigenic change and absolute humidity across subtypes were near zero (Fig. 6), with tight credible intervals (95% credible intervals: (−0.56, 0.27) for absolute humidity, (−0.50, 0.30) for antigenic change). Prior cases of an old variant given no antigenic change (95% credible intervals (−0.26, 0.85)), prior cases of all variants for non-first epidemics (95% credible intervals (−0.84, 0.33)) and rainfall during the epidemic (95% credible intervals (−0.68, 0.20)) also showed no strongly discernible effects, though with less posterior certainty. The model could not explain much of the variation in the data: the median-estimated standard deviation of log epidemic size about the expected log size is 0.77 (95% credible intervals (0.67, 0.90)). Since exp(0.77) is ~2.15, this implies that it is not unusual to see epidemics half or twice the expected incidence. The model estimated that the effects were very similar across subtypes (Supplementary Fig. 14, median-estimated SDs for the distribution of subtype-specific effect sizes about the overall mean effect size near zero, Supplementary Fig. 15). Only the effect of whether an epidemic was the first of the season showed meaningful heterogeneity: the model estimated that it is somewhat weaker for B/Vic than for other subtypes (Supplementary Fig. 14).

## Discussion

Based on city-level analyses of a subtyped and antigenically characterised influenza virus data set covering the five largest cities in Australia, we find that climate and antigenic novelty have limited effects on epidemic sizes. The results presented here suggest that, at least in temperate areas, epidemics are governed by factors other than host immunity at local scales, where global fitness advantages for new antigenic variants may not be realised. Conversely, competition for hosts among influenza virus types and subtypes has strong effects on local dynamics. The first virus subtype to establish above-baseline epidemic activity in a city and season typically dominates.

A recent study of fine-scale influenza epidemiology in Australia^23^ showed that there was substantial heterogeneity among Australian cities in the activity of influenza A and B viruses. Our subtyped and antigenically characterised data set allowed us to confirm that further heterogeneity exists at the level of antigenic variants. In particular, specific antigenic variants often cause large epidemics in some cities while not causing detectable activity in others.

While prior studies found that the onset of epidemics in the United States and France was preceded by a 2-week period of anomalously low absolute humidity^10,13^, we found no evidence for climatic effects when aggregating across the five Australian cities. Anomalous fluctuations in temperature and absolute humidity were sometimes positive, sometimes negative, but on average approximately zero. Importantly, the overall effect size reported by Shaman et al.^10^, after aggregating across all 48 contiguous states of the United States, was very small (with mean AH′ being ~−0.25 kg kg^−1^ or −0.21 g m^−3^, compared against 0 g m^−3^, the mean of the null distribution of historic wintertime values). About 55–60% of epidemics were preceded by negative AH′ values: a moderate increase upon the null hypothesis being a baseline of 50%.

Shaman et al.^10^ also found regional differences in the associations between fluctuations in absolute humidity and epidemic onset. Strong associations were found in the Southeastern United States but not in Western states. In Australia, there does not appear to be an aggregate effect at the country level, and there were no consistent patterns at the level of individual cities (Supplementary Fig. 2 and Supplementary Table 2). The small effect sizes and lack of consistency in climatic patterns across regions and cities in the United States and Australia may reflect the fact that climatic factors alone are unlikely to account for the differences in the patterns of influenza seasonality between temperate and tropical regions^26^.

Seasonal epidemic waves in the United States appear to begin in the Southern states, which have warmer and more humid climates^21,27^, casting some doubt on the role of low humidity as a trigger for influenza epidemics. Rather than acting as specific triggers, it is plausible that climatic factors are acting on longer timescales than the anomalous fluctuations reported by Shaman et al.^10^ to more generally enhance transmission and increase incidence^28^. However, in Australia, epidemic size does not appear to be strongly associated with the mean temperature or absolute humidity over the epidemic period.

Given the interest in influenza virus as a model system for phylodynamics of a pathogen that consists of multiple co-existing antigenic variants^29^, there is interest in understanding how competition between these related variants, typified by cross-immunity, shapes epidemiological dynamics. Studies have hypothesised that antigenic change should result in larger^16,30–32^ and earlier^18^ local epidemics, which exhibit greater spatiotemporal synchrony at the national level^19–23^. The sequential replacement of old antigenic variants by new ones is indicative that antigenicity and population immunity are important for the global-level phylodynamics of influenza viruses. In contrast, at the local level, we find for A/H3 and B/Vic viruses that neither antigenic change nor the accumulation of antigenic variant-specific immunity are strong drivers of epidemic size, though accumulating variant-specific immunity may moderately reduce the probability of successful epidemic initiation.

It is striking that individual antigenic variants of A/H3 and B/Vic viruses are capable of re-invading the same city multiple times over consecutive years, despite a lack of substantial antigenic change. A/H1pdm09 viruses had previously been shown to cause repeat epidemics without antigenic change^33,34^, but our study establishes that this occurs for multiple types and subtypes of human influenza. One possible explanation for the lack of evidence for the year-on-year depletion of susceptible hosts is that influenza virus infection often fails to confer strain-specific and effective immunity. In some individuals, antigenic seniority and existing immunity against previously encountered antigenic variants may suppress novel strain-specific antibody responses, leading to only modest specific protection against reinfection^35,36^. Similarly, vaccine trials suggest that multiple exposures can be required in order for children to become seropositive sufficiently to protect themselves^37^. Potentially, multiple natural infections may also be needed to confer protective immunity^38^, particularly in children^39^, who effectively form a non-depleting pool of susceptibles.

There may also be substantial and previously unaccounted heterogeneity in individual susceptibility towards the same virus strain. The notion that population-level strain-specific immunity to influenza viruses is monolithic may be an artefact of the single-infection ferret models typically used to estimate antibody-mediated protection. In humans, there is substantial individual-to-individual variation in the antigenicity of amino acid escape mutations for influenza haemagglutinin^40^. Such heterogeneity between individuals stems from their varied exposure histories to different influenza viruses. Unfortunately, age records for our data set were too incomplete to allow us to study age-specific heterogeneities in demographics, and attack rates between cities, and whether such patterns change over seasons.

Spatial and social connectivity structures among hosts in a city may also limit the spread of epidemics. Heterogeneous contact patterns between hosts can have a substantial impact on the resulting epidemiological dynamics^41,42^. Epidemics may be inherently frail processes: relatively minor human behavioural or environmental perturbations could prematurely terminate epidemics before they exhaust the pool of susceptible hosts, preserving a substantial number of susceptibles, and permitting subsequent epidemics of the same antigenic variant.

While our data set is substantially smaller (>450,000 vs 18,250 cases) than the one analysed by Geoghegan et al.^23^, and is thus more likely to be affected by noise in epidemic and surveillance processes, the differences between our findings and theirs highlight the importance of subtyping and antigenic characterisation, particularly for drawing conclusions about the effects of antigenic change. Geoghegan et al.^23^ had cautiously suggested, given only virus-type data, that the 2009, 2012 and 2014 influenza A virus epidemics in Australia exhibited greater spatiotemporal synchrony potentially due to the emergence of the novel A/H1pdm09 subtype in 2009 and novel A/H3 antigenic variants in 2012 and 2014. However, with further subtype resolution and antigenic characterisation, we find that the majority of influenza A activity in Adelaide and Melbourne in 2014 was attributable to A/H1pdm09, rather than the (antigenically novel) A/H3; in fact, there was no above-baseline A/H3 activity in Perth. The fact that different virus subtypes caused these apparently synchronous epidemics implies that the epidemic synchrony described by Geoghegan et al.^23^ was not due to the antigenic evolution or regional spread of a single virus strain.

Apart from competition between antigenic variants, previous epidemiological studies have hypothesised the existence of heterosubtypic competition where prior infection by a virus of one subtype is negatively associated with subsequent infection by a virus of another subtype^43,44^. In agreement with a previous US study of national-level ILI activity augmented with limited virus subtyping^45^, we also find evidence for a first-mover advantage and competition to infect hosts within a city, where the subtype or type that initiates above-baseline levels of activity first is most likely to have the largest epidemic of that season.

There are multiple caveats to our study that merit explicit consideration. The most important ones derive from our use of passive surveillance data that might not accurately reflect true underlying influenza virus activity. For example, surveillance intensity could plausibly vary between cities and years. While variation in surveillance efforts is evident among cities, there was no evidence of systematic increases or decreases in the number of laboratory-confirmed cases, or changes to surveillance practices within each city during the study period. Despite this, the longer duration of epidemics recorded after 2009 could be indicative of enhanced surveillance in the post-pandemic era: to mitigate this possibility, we repeated our analyses on the effect of antigenic change on epidemic size, splitting between pre- and post-pandemic eras and epidemic sizes normalising by their respective era-specific means. In either era, there was no consistent effect of antigenic change on epidemic size, with the caveat that splitting across eras reduced the number of observations in each era and thus our statistical power (Supplementary Fig. 18).

The intensity of surveillance could also vary over the course of an epidemic. For example, sentinel physicians could become more likely to submit samples for further testing as an epidemic unfolds, or conversely, testing could prematurely cease as facilities become overwhelmed with samples. Despite being unable to definitively rule out the former scenario, the latter is unlikely to affect our data. If reporting ceased after a certain number of samples had been tested, the distribution of epidemic sizes would be truncated, and each epidemic would be unlikely to have an exponentially declining tail. No such patterns exist in our data.

The intensity of surveillance could also potentially vary across subtypes and lineages. The mean age of infection for A/H3 is greater than influenza B^46^ viruses, and healthcare-seeking behaviour may differ between adults, parents with children and children. Furthermore, it is commonly thought that A/H3 virus infections result in more severe clinical presentations and greater risk of mortality^47^ than influenza B viruses, potentially resulting in differences in the likelihood of detection by a sentinel health practitioner, though this may not be the case (see ref. ^48^).

Another important caveat is that while we were able to include antigenic data in this study, these data were all derived from haemagglutination inhibition (HI) assays. HI assays do not measure virus antigenic changes that occur away from the receptor-binding site, and thus likely represent an incomplete picture of antigenic change. Reference viruses and sera used in the haemagglutination inhibition assays can also impact the interpretation of the assay readout, and the HI data used in this study were therefore treated with caution (see ‘Methods').

In this study, we attempted to identify associations between population susceptibility and epidemic incidence. Accurately quantifying the former is a complex challenge, so cumulative antigenic variant-specific epidemic incidence was used as a proxy, but that itself is subject to the limitations listed above. Besides natural infection, immunity can also be derived from vaccination, the contribution and effectiveness of which could not be determined due to a lack of temporally and geographically complete vaccination records over the study period. Regardless, we hypothesise that the impact of seasonal vaccination would be limited, particularly in the context of Australia, given the low uptake of vaccination^49^. Crucially, the uptake by children, who are important in driving local community transmission, is often below 10%^50^.

While our Bayesian multilevel model estimated negligible effects on epidemic size stemming from climatic factors and prior cases attributed to the same antigenic variant, the estimated credible intervals were not tight enough to rule out these effects conclusively (Fig. 6). However, our study suggests that climatic and antigenic factors are unlikely to be strong drivers of local influenza epidemiological dynamics. Indeed, the effects of these specific factors are dwarfed in magnitude by more generic epidemiological drivers: seasonality not directly captured by climate (measured by start date) and competition for hosts among subtypes (measured by whether an epidemic is the first of the season) (Fig. 6). We also find that even with all generic and specific factors considered, precise predictions of epidemic size remain difficult because of substantial noise in the local epidemic process.

Our Bayesian multilevel model for epidemic size avoids explicitly modelling underlying transmission processes, and may fail to fully capture the nature of the relationship (linear vs nonlinear) between transmission rates/R_0_ and the total cases in an epidemic. However, based on previous virus-transmissibility studies^7^, if climatic factors are strong drivers of epidemiological dynamics, we would expect the climatic variabilities observed in Australia to have a substantial impact on transmission rates, and produce detectable differences in epidemic size, but this is not the case.

Climatic drivers of seasonality and homosubtypic competition between virus antigenic variants are thought to be strong drivers of seasonal influenza epidemiology, but seasonal influenza virus epidemiological dynamics in major Australian cities appear to be more substantially shaped by other factors, particularly the establishment of sustained virus-transmission activity, and subsequent competition among virus types and subtypes. This implies that the time horizon for meaningful forecasting of epidemic subtype composition is very short (days to weeks), and forecasting efforts aimed at longer-term predictions will require further insights into the dynamics of virus introduction and epidemic establishment, and into the accumulation of population immunity to seasonal influenza viruses.

## Methods

### Australian surveillance data

Influenza viruses from Australia were collected by the WHO Collaborating Centre (WHOCC) for Reference and Research on Influenza in Melbourne, Australia. The Melbourne WHOCC receives a subset of influenza-positive clinical samples collected by various sentinel surveillance systems across Australia throughout the year. The samples in this study were typed, subtyped and antigenically characterised by haemagglutination inhibition assay to the vaccine reference vaccine strain in use at the time of sample collection.

The data set consists of 18,250 influenza-positive cases, collected between 2000 and 2015 in the city of Brisbane, the city of Perth, the state of South Australia, the city of Sydney and the state of Victoria. The breakdown at the subtype/lineage level is as follows: A/H3 (7661), A/H1sea (1410), A/H1pdm09 (3987), B/Vic (3021) and B/Yam (2171). All of these correspond specifically to individual cities, except for the data from Victoria and South Australia. As of June 2015, 75 and 78% of the inhabitants of the states of Victoria and South Australia resided in the cities of Melbourne and Adelaide, respectively. We therefore treated the Victoria and South Australia data as representative of city-level patterns in those two major cities.

All epidemic activity of all subtypes for the 2009 season was excluded from all analyses because of the 2009 A/H1N1 pandemic. Unsurprisingly, patterns of virus circulation during the pandemic were anomalous compared with typical seasonal influenza virus epidemics, and potentially distortive of the patterns we sought to characterise.

### Estimation of epidemic timing

The exact timing of interseasonal periods of sporadic activity and epidemic onset for each subtype is highly variable between years, even for individual cities, so it is necessary to determine the onset and end of each epidemic independently for each antigenic variant, season and city.

For each individual antigenic variant-specific time series, we used a Poisson count detection algorithm implemented in the Surveillance package in R^51,52^ to distinguish periods of sustained epidemic activity from a background of sporadic interseasonal activity. We assume that the start of the calendar year falls sometime within the interseasonal period, which is justified by the scarce number of cases observed during this time of the year, and the fact that it is summertime in Australia. Making no further assumptions on the exact duration and timing for the interseasonal period or epidemic onset, starting at the beginning of the year, successive 2-week periods *y*_*t*_ are evaluated using the number of cases in each of the *n*-preceding 2-week periods $\left\{ {y_{t - n},y_{t - n + 1}, \ldots, y_{t - 2},y_{t - 1}} \right\}$ as reference values for sporadic activity. These reference values are used to predict a threshold value *y*_*α*_: if the observed number of cases *y*_*t*_ exceeds the threshold *y*_*α*_, the focal 2-week period is marked as the 2-week period of epidemic onset.

The Poisson count model assumes that the reference values *y*_*t*_ are identically and independently Poisson distributed with a mean of *λ* (Eq. (1)). *λ* itself has a Gamma distribution as a prior (Eq. (2)). From Eqs. (1) and (2), the posterior predictive distribution is a negative binomial distribution (Eq. (3)).

1 $$y_i\sim Po(\lambda )$$

2 $$\lambda \sim Ga(\alpha ,\beta )$$

3 $${\mathrm{z}}|y_{t - n},y_{t - n + 1}, \ldots ,y_{t - 2},y_{t - 1}\sim NegBin\left( {\alpha + \mathop {\sum }\limits_{i = 1}^n y_i,\frac{{\beta + n}}{{\beta + n + 1}}} \right)$$

The threshold value *y*_*α*_ can then be calculated using quantile parameter *α*, where *y*_*α*_ is the smallest value that satisfies Eq. (4).

4 $$p\left( {y \le y_\alpha } \right) \ge 1 - \alpha$$

We used the same algorithm to identify the end of an epidemic. Starting at the end of the year, successive 2-week periods, in the backward direction, are evaluated using the number of cases in each of the *n* following the 2-week period as reference values $\left\{ {y_{t + 1},y_{t + 2}, \ldots ,y_{t + n - 1},y_{t + n}} \right\}$.

During interseasonal periods, where there were often many 2-week periods reporting no cases, an isolated 2-week period with sporadic activity can be misconstrued as the onset of an epidemic. To reduce the impact of outliers in the time series and increase specificity of the detection algorithm, we first applied the 4253H, twice nonlinear data-smoothing algorithm^53^, which is a compound smoother consisting of multiple running medians.

We tested a variety of *n-* and *α*-parameter values, and chose *n* = 3 and *α* = 0.12 for the analyses presented in the text as a good compromise between sensitivity and specificity in the identification of all of the epidemics within the time series and their individual onset and end timings, which were confirmed by visual inspection. The results of these analyses are also robust to alternative parameter values and corresponding changes to the sensitivity and specificity of the Poisson count detection algorithm (see Supplementary Discussion for sensitivity analyses).

Aggregation of cases by 2-week periods was deemed necessary, to smoothen the time series in light of the relatively low number of cases within the data set; this relatively long timescale could however potentially obscure fluctuations in weather that occur at shorter scales. Whilst weekly time series were appreciably noisier, we found a high degree of correspondence in the estimated epidemic onset and end timings with values calculated from data aggregated by 2-week periods: indeed, our results were robust to aggregation by week (see Supplementary Discussion for sensitivity analyses).

We deemed an antigenic variant to have failed to cause an epidemic if, within a season, the algorithm was unable to define an epidemic period; we confirmed all putative failures by inspection of the raw time series. Once the epidemic period was defined, the size of an epidemic per antigenic variant was calculated using the estimated resident population for that particular year and city.

### Normalisation of epidemic incidence

For each epidemic, the incidence of laboratory-confirmed cases per million people was calculated from the number of raw counts. Given the positive skew in the distribution of epidemic incidences, individual incidence values were log-transformed. To enable comparisons within subtypes, we needed to account for potential differences in surveillance intensity, and normalise values between cities: we subtracted off the overall city-specific mean log-transformed incidence from each individual value. Although the apparent heterogeneity in the effect of antigenic change and prior immunity between subtypes suggests that data should be stratified by subtype, we repeated our analyses with data aggregated and normalised across subtypes in order to increase statistical power. Individual log-transformed values for each epidemic were instead transformed by subtracting off the overall city- and subtype-specific mean of the log-transformed values.

### Virus antigenic characterisation by haemagglutination inhibition assay

For our analyses, we defined an antigenic variant as in Smith et al.^14^, where an antigenic variant is sufficiently different from preceding variants to warrant an update of the seasonal influenza virus vaccine. To this end, our analyses only accounted for major antigenic changes, and did not account for the possibility of small or gradual antigenic changes (neither of which are well studied for seasonal influenza viruses).

The haemagglutination inhibition (HI) assay data used in this study only compared the test virus and the then current reference vaccine strain to assess whether or not viruses had changed antigenically. However, this comparison to a single reference point is potentially problematic, given that new Southern Hemisphere’s influenza vaccine composition recommendations are made every September. This is usually after the end of the influenza season in Australia, and may lead to misidentification during antigenic characterisation of submitted samples during the preceding season where samples containing a novel antigenic variant may have been tested with sera raised against its predecessor variant. To ameliorate this potential source of bias, we compared the antigenic characterisation data against phylogenetic data. This comparison revealed two instances for A/H3 viruses where the reference strain comparison by HI was misleading regarding the antigenic composition of an epidemic. There were a substantial number of laboratory-confirmed cases attributable to A/H3/Fujian/411/2002-like viruses in 2004, but phylogenetic analyses of sequences dated 2004 show that the Fujian/411/2002-like viruses had already been replaced by the novel California/7/2004 variant viruses. Similarly, in 2005, a substantial number of samples initially identified as A/H3 California/7/2004-like viruses were phylogenetically in the new A/Wisconsin/67/2005 variant group.

To account for the likelihood of misidentification due to delays in updating nomenclature, we assumed that all A/H3 cases in 2004 were California/7/2004-like, and in 2005 were Wisconsin/67/2005-like antigenic variants. Additional analyses were also carried out with the raw data set without these corrections (see Supplementary Figs. 9–13 and Supplementary Table 6), and lead to no significant or substantive differences to our findings.

### Demographic data

We retrieved estimated resident populations for Adelaide, Brisbane, Melbourne, Perth and Sydney on 30 June of each year from 2000 to 2015 from the Australian Bureau of Statistics (http://stat.abs.gov.au/).

### Climate data

For each of the five cities, we compiled the mean temperature (°C) and relative humidity (%) from TuTiempo (https://en.tutiempo.net/), and calculated the mean absolute humidity (g m^−3^) for each 2-week period from 1985 to 2015. For each of the 26 2-week periods of the calendar year, we calculated 31-year mean temperature $\bar T$ and absolute humidity $\overline {AH}$ values (see Eqs. (5) and (6) below).

### Testing the statistical significance of anomalous absolute humidity and temperature

Following the method presented by Shaman et al.^10^, we calculated local anomalous *T*′ and absolute humidity *AH*′ values for each city, and 2-week period of the year from 2000 to 2015. For each 2-week period, *T*′ and *AH*′ are defined as the deviation in observed temperature *T* and actual absolute humidity *AH* from their 31-year mean values, $\bar T$ and $\overline {AH}$, respectively (Eqs. (5) and (6)).

5 $$T^{\prime} = T - \bar T$$

6 $$AH^{\prime} = AH - \overline {AH}$$

Following Shaman et al.^10^, we generated a synthetic distribution of wintertime climatic values by bootstrap sampling. In order to maintain the sampling structure and control for anomaly variability among the cities, 15 n-week continuous blocks were randomly sampled from 1 April–31 August, 1985–2015 for each of the five cities. These 75 samples were then averaged to produce a mean *T*′ and *AH*′ value. This was repeated 100,000 times to produce a bootstrapped distribution of average values. The statistical significance for the mean *T*′ and *AH*′ values derived from the 75 empirically observed earliest-in-the-season epidemics was then calculated non-parametrically, by determining the quantile for the observed values within the bootstrap distributions. This bootstrap was repeated at the city level to see if there were geographical differences with individual bootstrap distributions that were created for each city.

We also evaluated whether or not epidemic onset is associated with climatic fluctuations that are anomalous for that particular time of the year. By definition, for any given 2-week period of the year, the 31-year mean for *T*′ and *AH*′ is 0. We used a Wilcoxon one-sample test to assess whether there were reductions in climatic values in the observed set of *T*′ and *AH*′ values in each of the 2-week blocks preceding the onset of the earliest epidemic of the season.

### Bayesian hierarchical regression

To estimate reasonable bounds on the possible effects of climate and antigenicity on epidemic size, we used a Bayesian hierarchical model that partially pooled effect-size estimates across subtypes, increasing the capacity to detect any potential effects without assuming a priori that effects should be the same across different subtypes. We fit the model using Markov Chain Monte Carlo (MCMC) with Stan^54^ and its R interface rstan^55^; Stan implements a no-u-turn sampler (NUTS)^54^. All data and code needed to reproduce the analysis and figures are provided in the project Github repository, along with directions in a README file.

In the model notation that follows, the symbol “~” is a “sampling statement”; it denotes that a random variable is distributed according to the given distribution. Normal distributions are parameterised as Normal(mean, standard deviation), generalised Student-T distributions are parameterised as Student-T(degrees of freedom, location and scale). Positive-constrained normal distributions (Half-Normal) are parameterised as Half-Normal(mode, standard deviation).

We predicted log incidence minus city- and subtype-specific mean log incidence as a function of the following predictor variables:

*X*_*1*_: whether the epidemic was the first epidemic for an antigenic variant in the city (binary, yes or no)

*X*_*2*_: cumulative prior incidence of the antigenic variant (measured as log(total prior cases/city- and subtype-specific mean cases per epidemic))

*X*_*3*_: mean absolute humidity during the epidemic, from the start to end date of the epidemic (measured as fortnight of the year)

*X*_*4*_: start date of the epidemic (measured as fortnight of the year)

*X*_*5*_: whether the epidemic was the earliest epidemic (of any subtype) in the city that year (binary, yes or no)

*X*_*6*_: the cumulative amount of influenza activity (of any subtype) in the city that year prior to the epidemic

*X*_*7*_: mean rainfall during the epidemic, from the start to end date of the epidemic (measured as fortnight of the year).

We omitted mean epidemic temperature as a predictor as it was highly collinear with absolute humidity. Any observed large effect of absolute humidity could therefore theoretically have been attributable to temperature, though in practice we estimated an effect near zero for absolute humidity.

We made a linear prediction of an epidemic’s normalised size given its values for ***X*** = (*X*_*1*_*,…,X*_*7*_). Effect sizes *b*_*i*_ for each predictor *X*_*i*_ were subtype-specific, with *b*_*ij*_ denoting the effect of variable *i* for subtype *j*. We also estimated subtype-specific intercepts *a*_j_.

We included cumulative antigenic variant activity and prior activity in the year only for old antigenic variants and epidemics that were not first of the year, respectively, that is, as interaction terms with one minus the corresponding binary variables. So the predicted mean- centred log size *<y*_*k*_ > of an epidemic of subtype *j* is given by Eq. (7), where X_ik_ denotes the value of X_i_ for epidemic *k*. Following Gelman^51^, we mean-centred and scaled continuous predictors so that effect sizes b would be directly comparable between binary and continuous predictors.

7 $$\langle y_k \rangle \, =	\, a_j + b_{1j}X_{1k} + b_{2j}X_{2k}\left( {1 - X_{1k}} \right) + b_{3j}X_{3k} + b_{4j}X_{4k} + b_{5j}X_{5k} \\ 	+ b_{6j}X_{6k}\left( {1 - X_{5k}} \right) + b_{7j}X_{7k}$$

We assumed that observed normalized log epidemic sizes y_k_ were normally distributed about their predicted log sizes <y_k_> with an unknown, estimated standard deviation *σ*_*y*_ (Eq. (8)):

8 $$y_k\sim {\mathrm{Normal}}( \langle y_k \rangle ,\sigma _y)$$

We assumed that subtype effect sizes *b*_*ij*_ for each predictor *i* and subtype *j* were normally distributed about a general mean effect size *<b*_*i*_ > , with an unknown, estimated predictor-specific standard deviation *σ*_*bi*_ (Eq. (9)):

9 $$b_{ij}\sim {\mathrm{Normal}}( \langle b_i \rangle ,\sigma _{bi})$$

Likewise, we assumed that intercepts *a*_*i*_ were normally distributed about a mean intercept *<a* > with an unknown, estimated standard deviation *σ*_a_ (Eq. (10)).

10 $$a_i\sim {\mathrm{Normal}}( \langle a \rangle ,\sigma _a)$$

We assumed that predictor-specific effect-size standard deviations *σ*_*bi*_ were half-normally distributed with mode 0 and an unknown, estimated standard deviation *σ*_*b*_ (Eq. (11)).

11 $$\sigma _{bi}\sim {\mathrm{Half-Normal}}(0,\sigma _b)$$

We placed weakly informative^56^ positive-constrained half-normal priors on the intercept, effect size and error-term standard deviations *σ*_*a*_, *σ*_*b*_ and *σ*_*y*_ (Eqs. (12–14)). Weakly informative priors rule out biologically or mathematically implausible parameter values while allowing data rather than assumptions to inform inferences regarding plausible values.

12 $$\sigma _a\sim {\mathrm{Half}} - {\mathrm{Normal}}\left( {0,0.5} \right)$$

13 $$\sigma _b\sim {\mathrm{Half}} - {\mathrm{Normal}}\left( {0,1} \right)$$

14 $$\sigma _y\sim {\mathrm{Half}} - {\mathrm{Normal}}\left( {0,1} \right)$$

We placed a weakly informative Gaussian prior on the mean intercept *<a*> (Eq. (15)) and a weakly informative Student-T prior on the mean effect sizes *<b*_*i*_> (Eq. (16)):

15 $$\langle {\mathrm{a}}\rangle \sim {\mathrm{Normal}}\left( {0,1} \right)$$

16 $$\langle b_j \rangle \sim {\mathrm{Student}} - {\mathrm{T}}\left( {3,0,2.5} \right)$$

The intercept prior was based on the degree of variation in the normed outcome variable to cover it while ruling out intercepts much larger or smaller than the largest and smallest observations. The effect-size prior was based on a recommendation for weakly informative regression effect-size priors (for scaled predictors) from the Stan prior recommendation wiki (https://github.com/stan-dev/stan/wiki/Prior-Choice-Recommendations).

We ran four MCMC chains, each with a 1000-step sample warmup period followed by 1000 saved posterior samples, for a total of 4000 posterior draws. We verified convergence by inspecting trace plots, and confirming that all parameters had sufficiently low Rhat values (all Rhat < 1.005) and sufficiently large effective sample sizes (all neff >16% of total sample size). We visualised posteriors as quantile dotplots^57^ to aid in visual estimation of distributions.

### Reporting summary

Further information on research design is available in the Nature Research Reporting Summary linked to this article.

## Supplementary information

Supplementary Information

Reporting Summary

## Data Availability

All of the data for these statistical analyses and models are available at the following Github repository: https://github.com/edwardkslam/australian_seasonal_flu.

## Source Data

Source Data

## Code availability

Code developed for these statistical analyses and models is available at the following Github repository: https://github.com/edwardkslam/australian_seasonal_flu.
