# Supplementary material for: The impact of climate and antigenic evolution on seasonal influenza virus epidemics in Australia
Source: Nat Commun. 2020 Jun 2;11:2741. doi: 10.1038/s41467-020-16545-6 (PMC7265451; doi:10.1038/s41467-020-16545-6)
Supplement: Supplementary file 1 — Supplementary Information [file 41467_2020_16545_MOESM1_ESM.pdf]

Supplementary Information

The impact of climate and antigenic evolution on seasonal influenza virus epidemics in Australia

Lam et al.

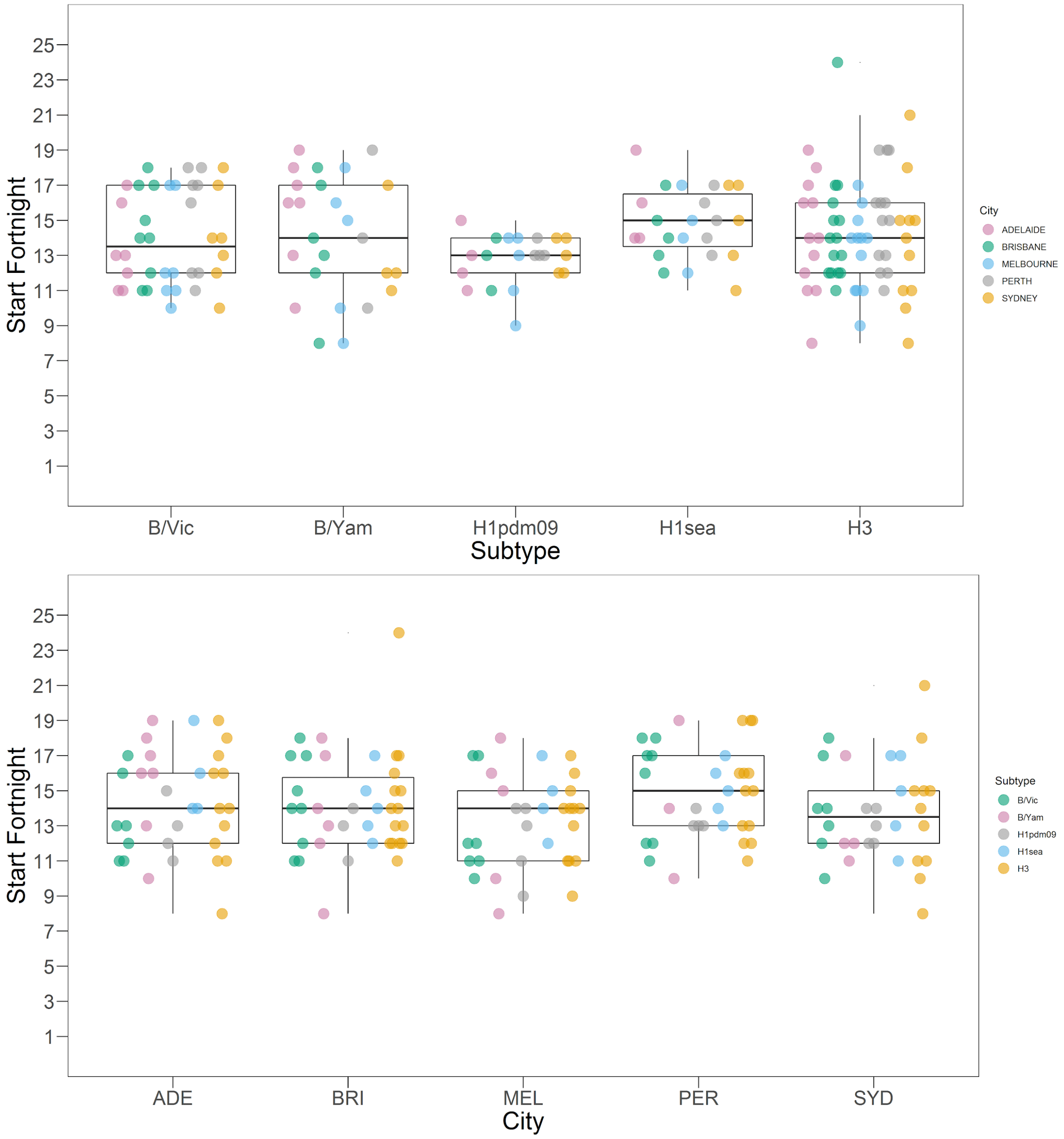

Supplementary Figure 1: Comparison of epidemic onset timing among subtypes and among cities.

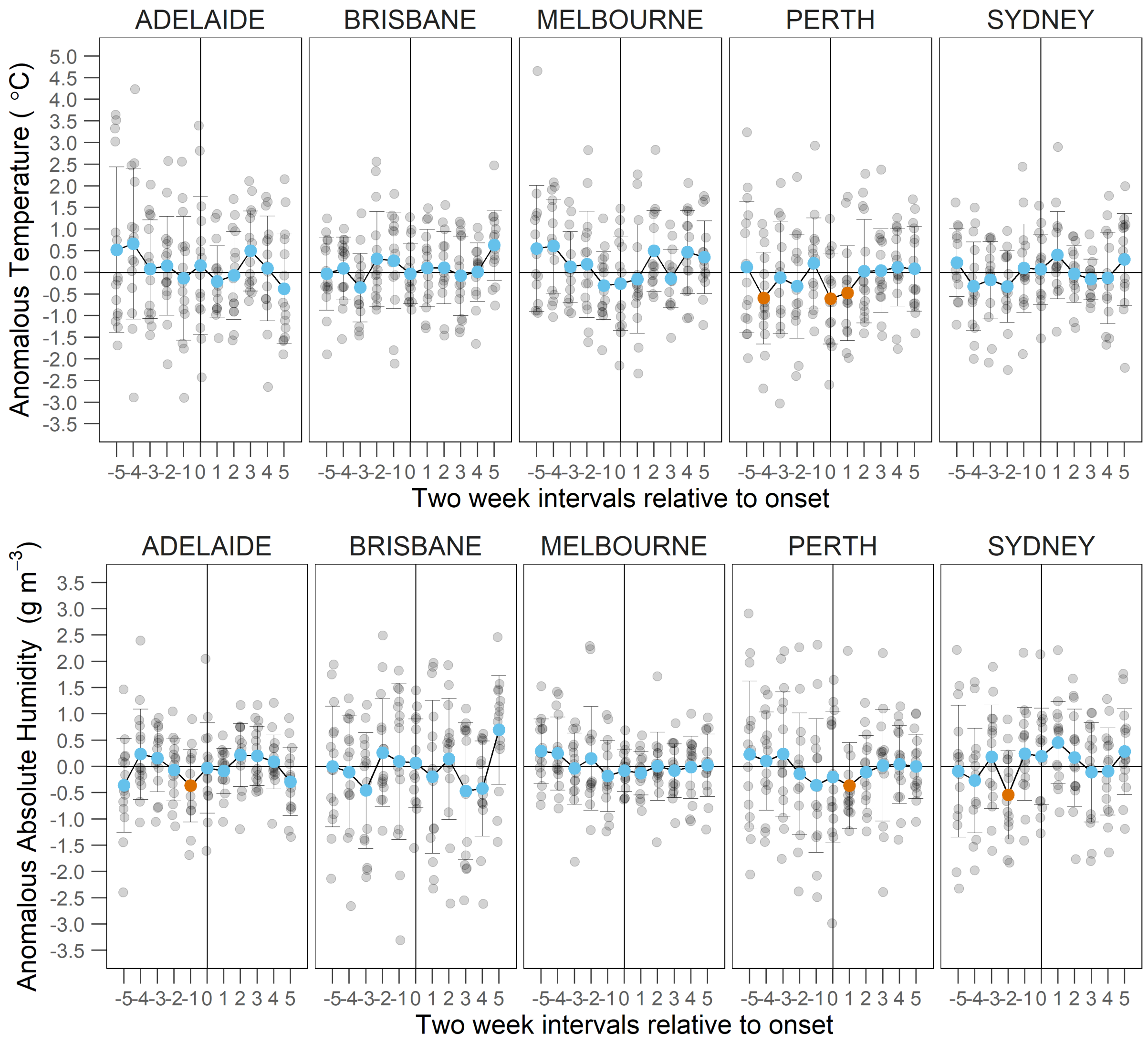

Supplementary Figure 2: Climatic conditions around epidemic onset. (**a**) Anomalous temperature T' and (**b**) absolute humidity AH' prior to and after epidemic onset for each of all five cities. Epidemic onset is marked by the vertical line at 0. For the earliest onset epidemic in each season and city (15 epidemics per city), T' and AH' for each time point are represented by grey points: a point below the horizontal line denotes that the value is lower than the 31 year city-specific mean. Blue points show the mean T' and AH' for that two week period for all epidemics within the study period in a particular city. Time periods with statistically significantly ($p<0.05$) reductions in mean T' or AH' from the 31-year average are shown in orange. In the two-week period immediately prior to epidemic onset, there is a statistically significant reduction in AH' of 0.366g m^{-3} in Adelaide ($p=0.021$, Wilcoxon one-sample test), which is roughly equivalent to a 3.11% reduction in relative humidity. This result was not statistically significant after correcting for multiple testing (Holm correction).

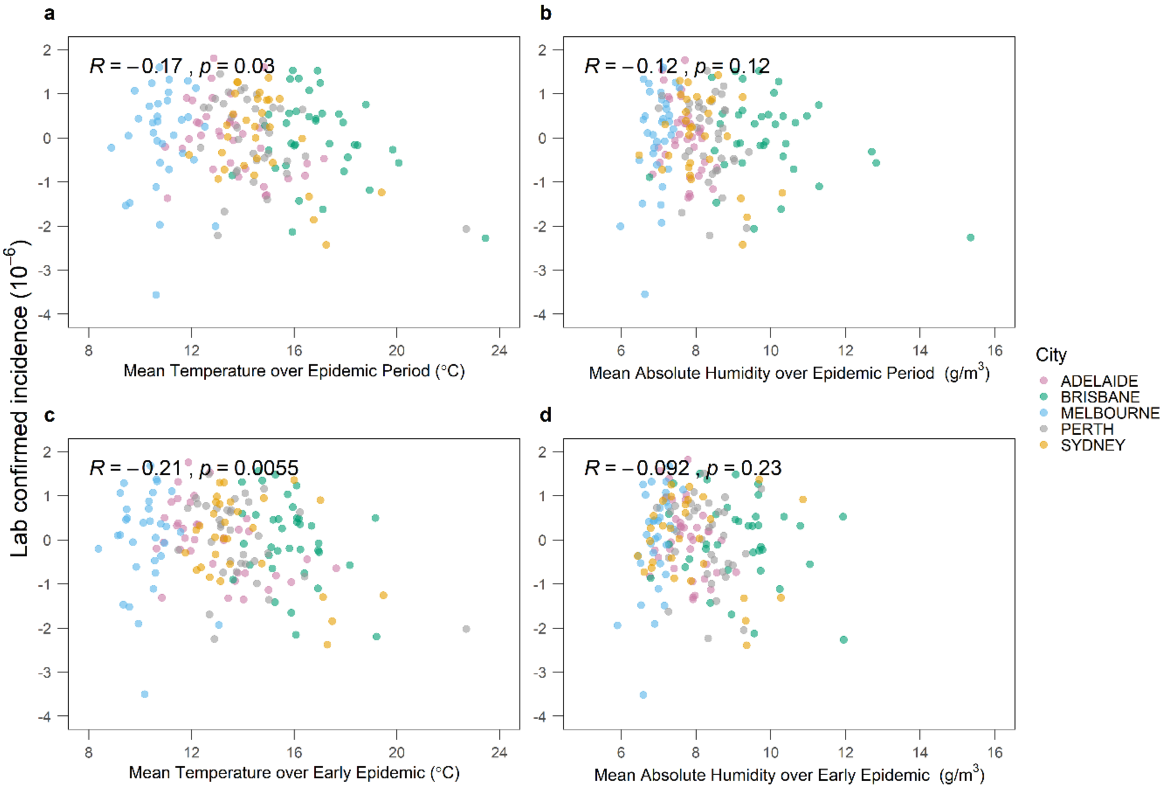

Supplementary Figure 3: Effect of climatic factors on epidemic incidence. The relationship between epidemic incidence and (**a,c**) the mean temperature and (**b,d**) absolute humidity values over the entire epidemic period (**a,b**) and the early epidemic (defined as period from the onset to the peak of an epidemic; Panels **c,d**). Incidence for individual epidemics were log transformed and subtracted by the city- and subtype- specific mean of log incidence, to allow for comparison across cities and subtypes. The distribution of mean climatic values displays segregation by city, reflecting underlying differences between climatic regions. Whilst there were weak negative associations between the size of an epidemic and mean temperature over the entire epidemic period or just the period from epidemic onset to peak, the high variability in epidemic sizes observed over a large range of climatic conditions and lack of overall, as well as within-city, trends suggest that climatic factors have limited and noisy effects on epidemic size.

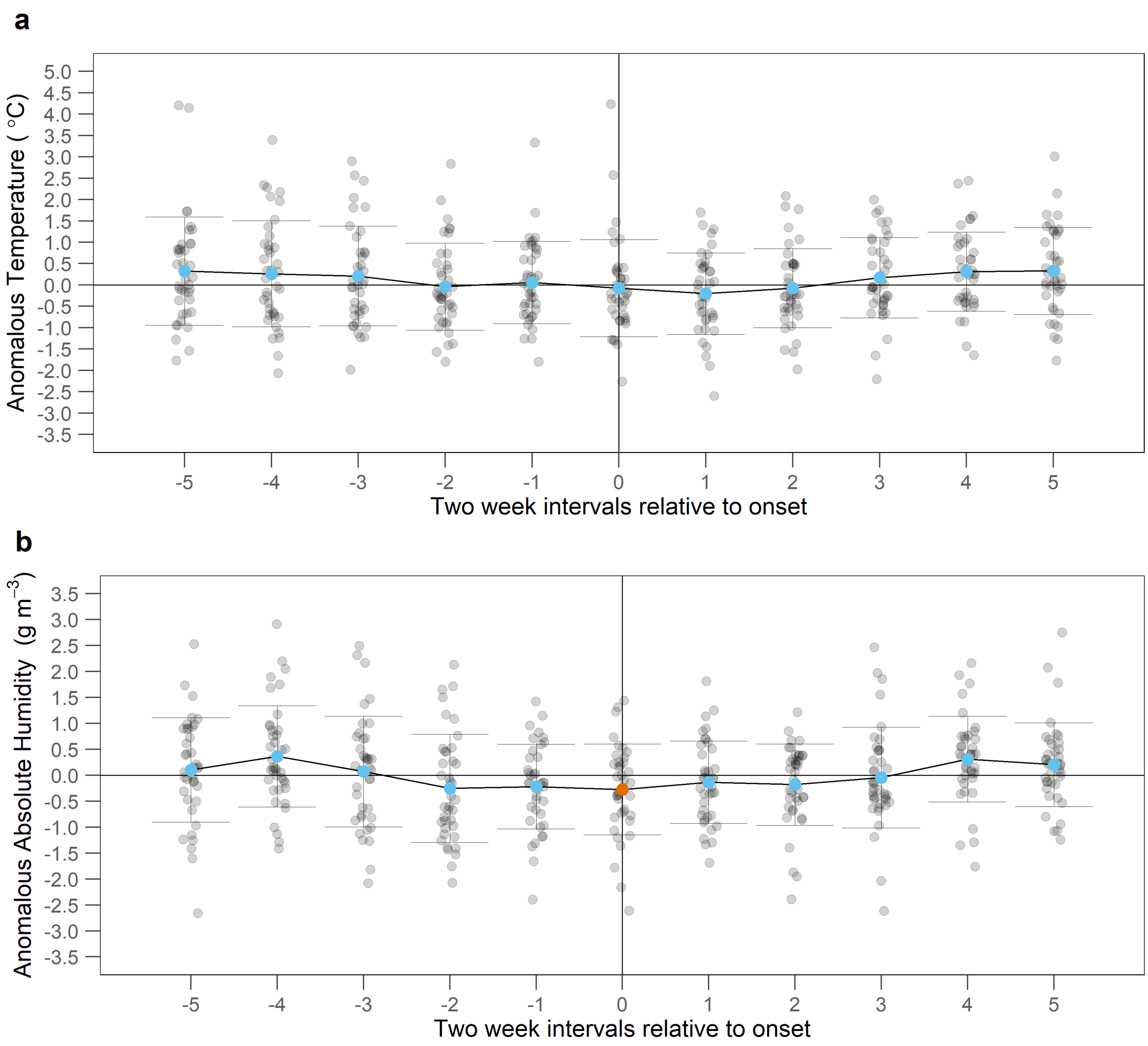

Supplementary Figure 4: Robustness of climatic factor analyses. Utilising only the timing estimates by Geoghegan et al.[23], we assessed if more generally, the onset of influenza A epidemic activity in the seasons from 2007 to 2015 was preceded by periods of anomalous climatic conditions; see Supplementary Discussion. (**a**) Anomalous temperature T’ and (**b**) absolute humidity AH’ prior to and after epidemic onset across all five cities. Epidemic onset is marked by the vertical line at 0. For the earliest onset epidemic in each season and city (8 years x 5 cities = 40 epidemics), T' and AH’ for each time point are represented by grey points: a point below the horizontal line denotes that the value is lower than the 31 year city-specific mean. Blue points show the mean T' and AH’ for that two week period for all epidemics within the study period. Time periods with statistically significantly ($p<0.05$; Wilcoxon one-sample test) reductions in mean T' or AH’ from the 31-year average are shown in orange.

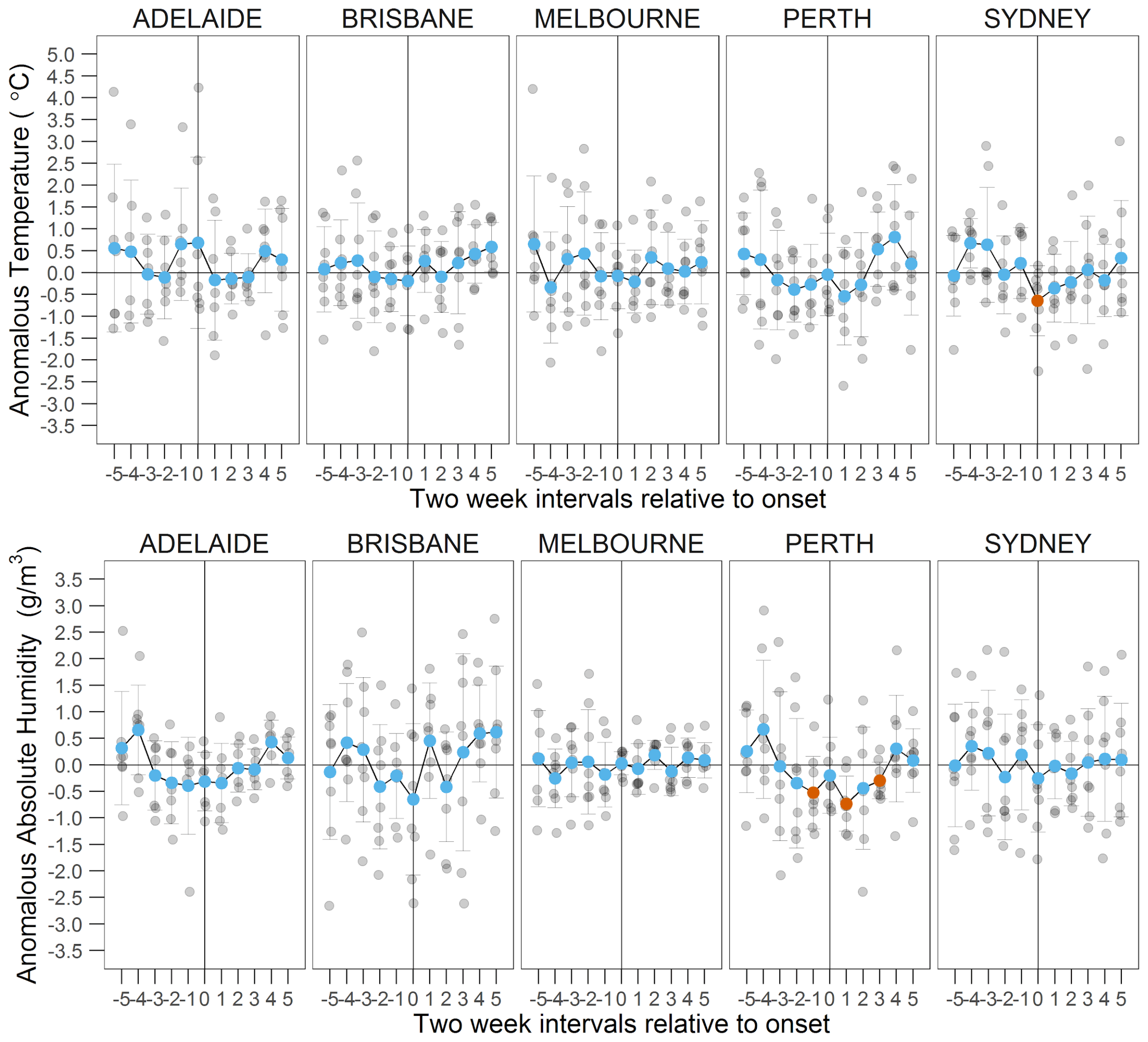

Supplementary Figure 5: Robustness of climatic factor analyses. Utilising only the timing estimates by Geoghegan et al.[23], we assessed if more generally, the onset of influenza A epidemic activity in the seasons from 2007 to 2015 was preceded by periods of anomalous climatic conditions; see Supplementary Discussion. (**a**) Anomalous temperature T’ and (**b**) absolute humidity AH’ prior to and after epidemic onset for each of all five cities. Epidemic onset is marked by the vertical line at 0. For the earliest onset epidemic in each season and city (15 epidemics per city), T' and AH’ for each time point are represented by grey points: a point below the horizontal line denotes that the value is lower than the 31 year city-specific mean. Blue points show the mean T' and AH’ for that two week period for all epidemics within the study period in a particular city. Time periods with statistically significantly ($p<0.05$) reductions in mean T' or AH’ from the 31-year average are shown in orange. Time periods with statistically significantly ($p<0.05$) reductions in mean T' or AH’ from the 31-year average are shown in orange. In the two-week period immediately prior to epidemic onset, there is a statistically reduction in AH’ of 0.522g m^{-3} in Perth ($p=0.039$, Wilcoxon one-sample test), which is roughly equivalent to a 3.13% reduction in relative humidity. This result was not statistically significant after correcting for multiple testing (Holm correction).

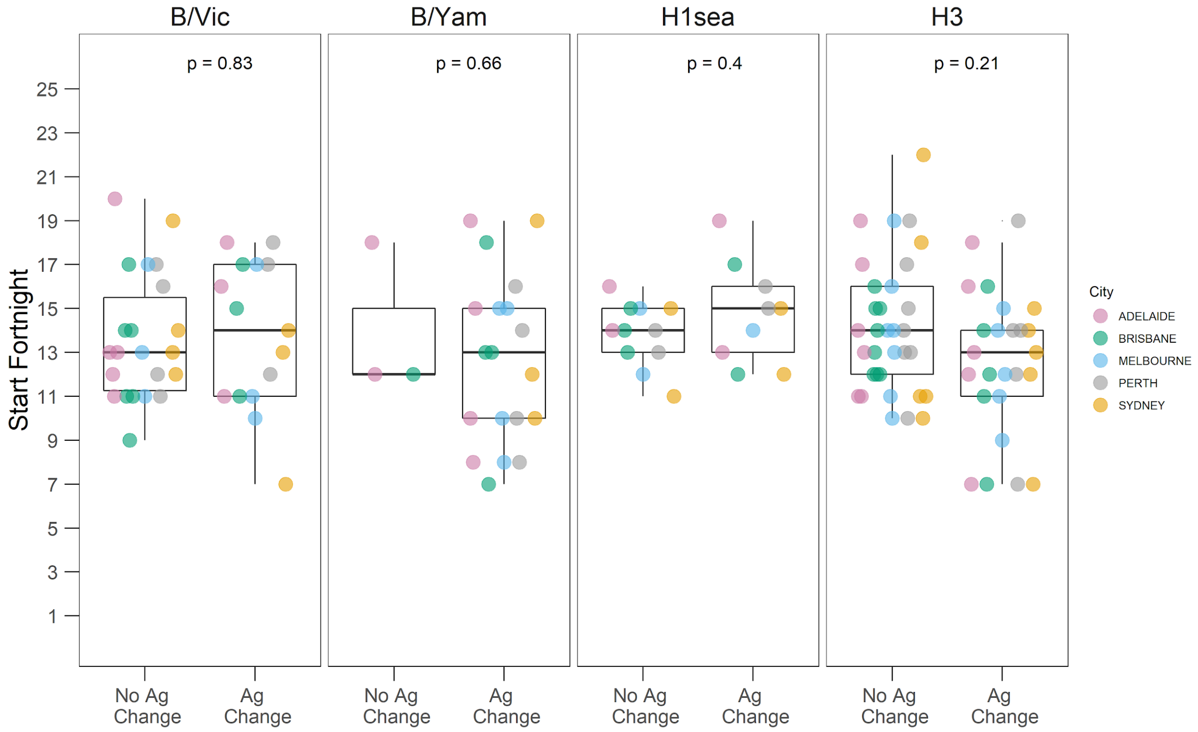

Supplementary Figure 6: Effect of antigenic change on epidemic onset timing. Epidemic onset timings were compared between seasons associated with and without the epidemic level circulation of a new major antigenic variant. *p* values are from Wilcoxon two sample tests (n = 37, 26, 22 and 63 for B/Vic, B/Yam, A/H1sea and A/H3 respectively). Box plots show the median, first and third quartile values, as well as overall range. Each point corresponds to one epidemic in a city and the box plots show the median, first and third quartile values, and range.

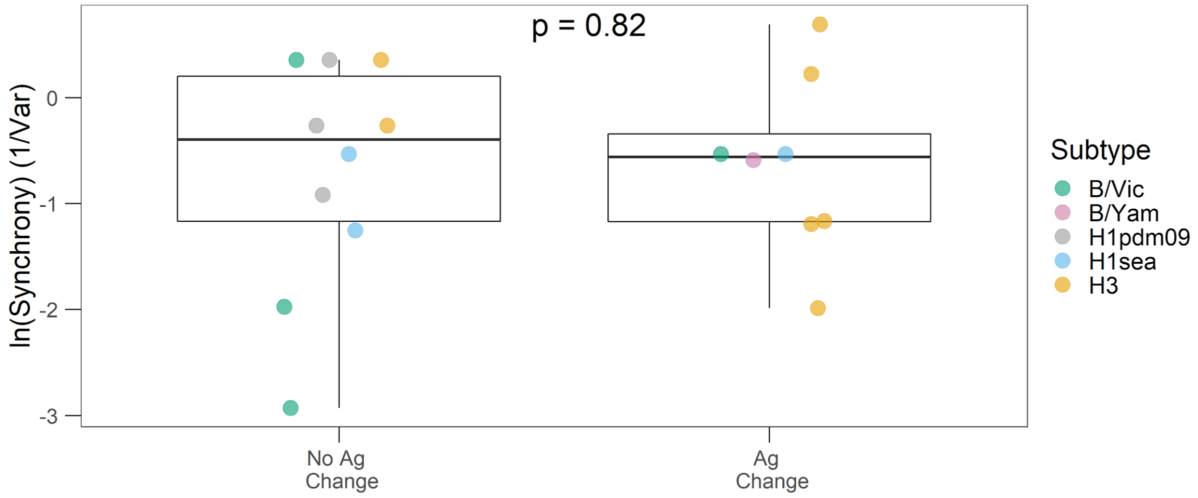

Supplementary Figure 7: Effect of antigenic change on the spatio-temporal synchrony of epidemics. Epidemic synchrony was compared between seasons associated with and without the epidemic level circulation of a new major antigenic variant. Synchrony is quantified as the reciprocal of the variance in onset timings for seasons, where epidemic activity for an antigenic variant was present in all five cities. *p* values are from Wilcoxon two sample tests (n = 18). Box plots show the median, first and third quartile values, as well as overall range.

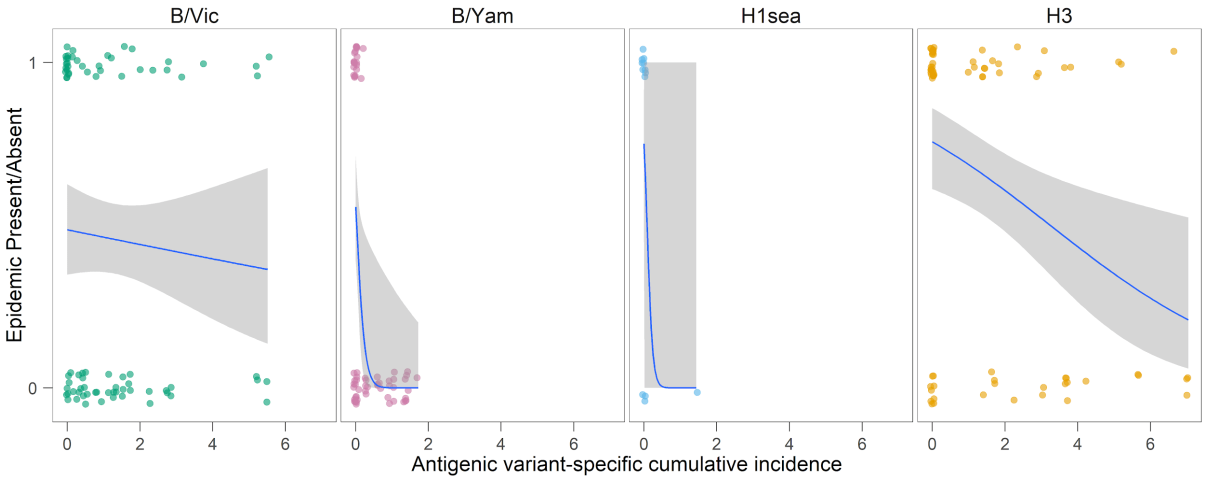

Supplementary Figure 8: Effect of prior immunity on the probability of successful epidemic initiation. For each antigenic variant, we examined whether epidemic levels of activity were present or absent in each of the seasons from its initial detection to its replacement by the next variant. . Antigenic variant-specific cumulative incidence was measured relative to the city-specific mean epidemic size, where 1 is equivalent to the mean epidemic incidence. Binary logistic regression models were fitted for each subtype (n = 81, 65, 13 and 72 for B/Vic, B/Yam, A/H1sea and A/H3 respectively). The 95% confidence interval is denoted by the grey shaded area. See Supplementary Table 5 for OR from the binary logistic regressions.

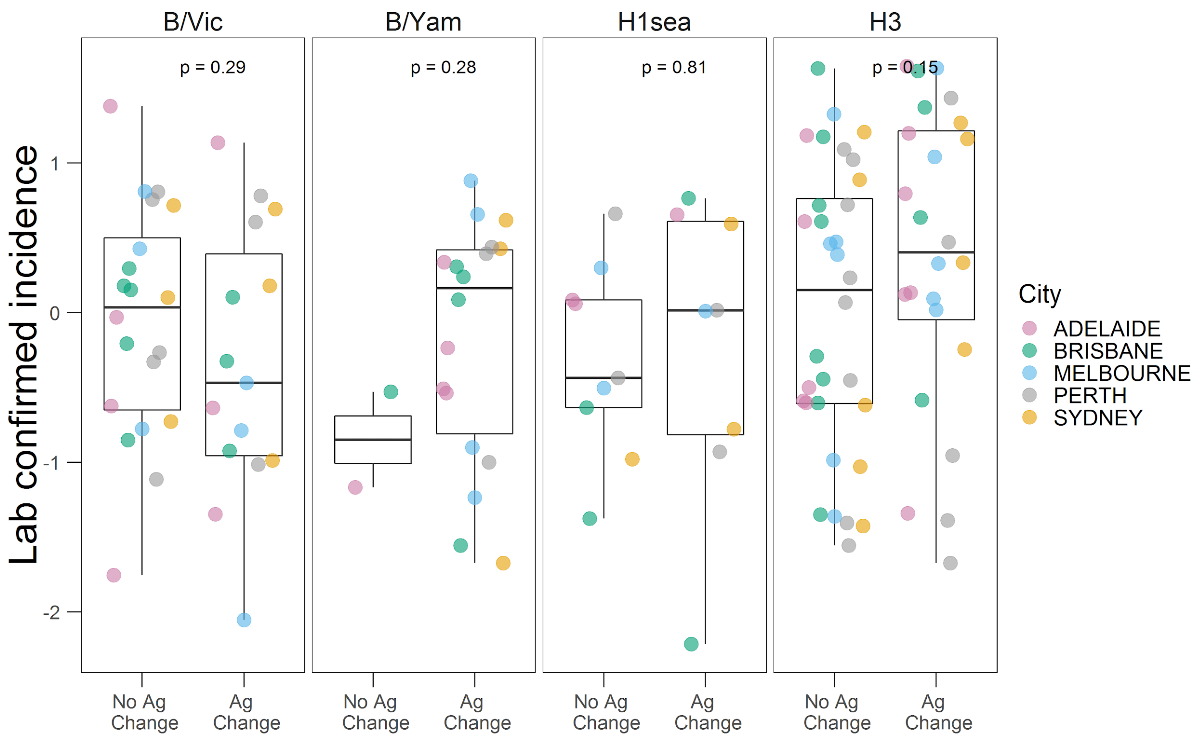

Supplementary Figure 9: Robustness of analyses of the effect of antigenic change on epidemic incidence, towards potential antigenic characterisation errors. Analyses as part of main text (Figure 3) attribute all A/H3 cases in 2004 to California/7/2004 and in 2005 to A/Wisconsin/67/2005 antigenic variants, due to delays in updating vaccine strain nomenclature. Here, we make no such assumptions. Epidemic incidence were compared between seasons associated with and without the epidemic level circulation of a new major antigenic variant. Within each subtype, incidence for individual epidemics were log transformed and subtracted by the city-specific mean of log incidence, to allow for comparison between cities. *p* values are from Wilcoxon two sample tests (n = 37, 26, 22 and 63 for B/Vic, B/Yam, A/H1sea and A/H3 respectively). Each point corresponds to one epidemic in a city and the box plots show the median, first and third quartile of the transformed values, and range.

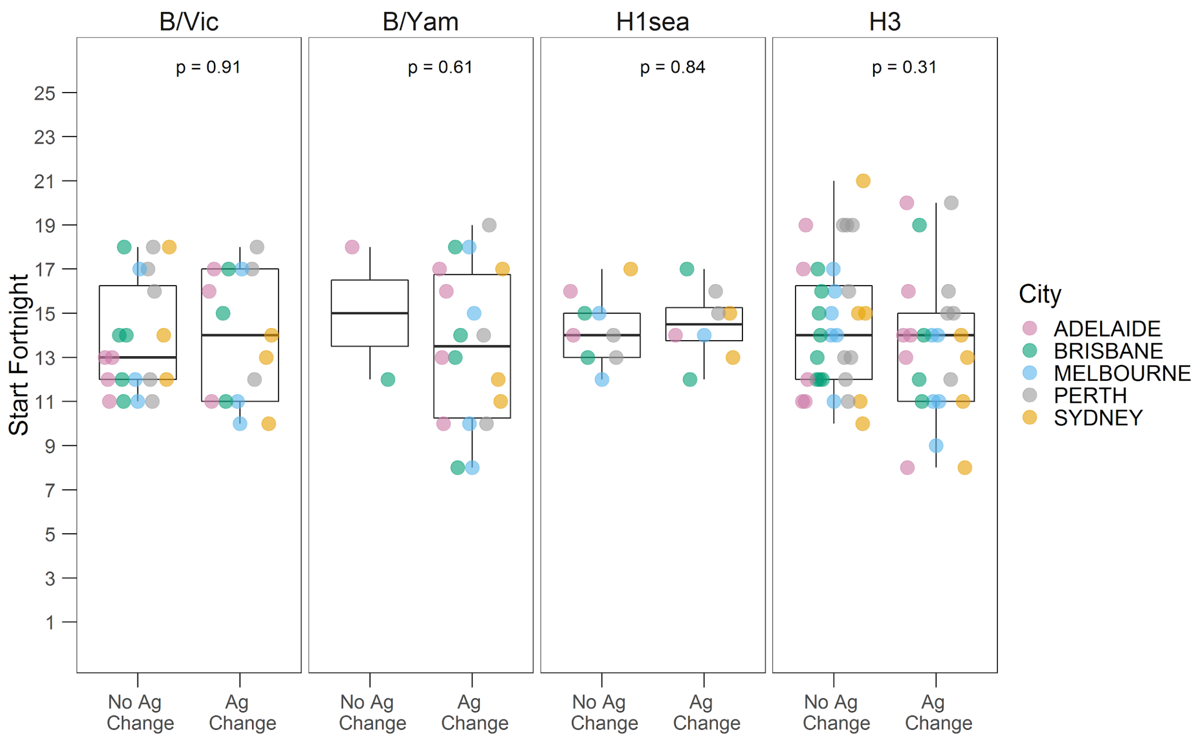

Supplementary Figure 10: Robustness of analyses of the effect of antigenic change on epidemic onset timing, towards potential antigenic characterisation errors. Analyses as part of main text (Supplementary Figure 6) attribute all A/H3 cases in 2004 to California/7/2004 and in 2005 to A/Wisconsin/67/2005 antigenic variants, due to delays in updating vaccine strain nomenclature. Here, we make no such assumptions. Epidemic onset timing was compared between seasons associated with and without the epidemic level circulation of a new major antigenic variant. *p* values are from Wilcoxon two sample tests (n = 37, 26, 22 and 63 for B/Vic, B/Yam, A/H1sea and A/H3 respectively). Box plots show the median, first and third quartile values, as well as overall range. Each point corresponds to one epidemic in a city and the box plots show the median, first and third quartile values, and range.

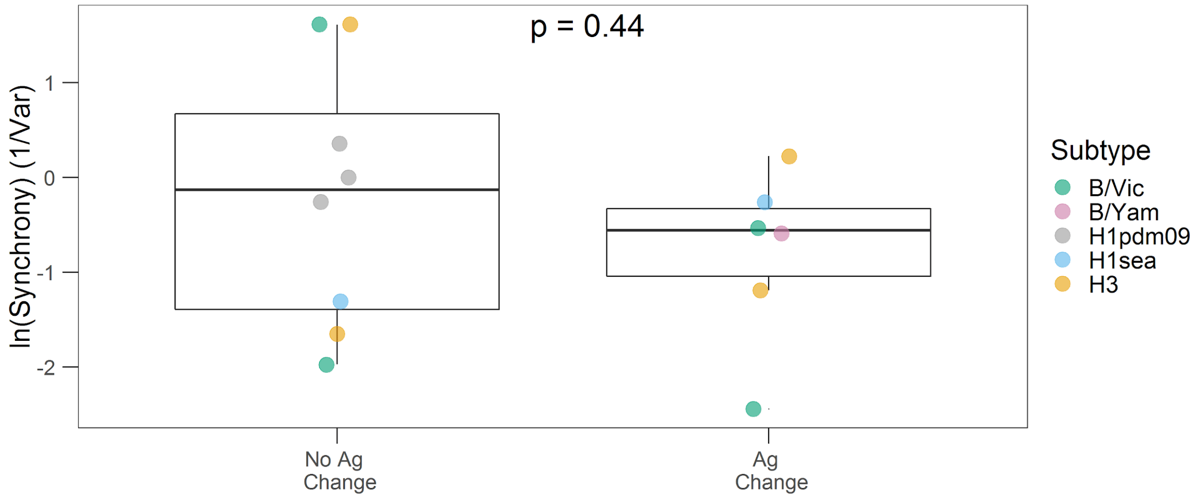

Supplementary Figure 11: Robustness of analyses of the effect of antigenic change on the spatio-temporal synchrony of epidemics, towards potential antigenic characterisation errors. Analyses as part of main text (Supplementary Figure 7) attribute all A/H3 cases in 2004 to California/7/2004 and in 2005 to A/Wisconsin/67/2005 antigenic variants, due to delays in updating vaccine strain nomenclature. Here, we make no such assumptions. The spatio-temporal synchrony of epidemics is compared between seasons associated with and without the epidemic level circulation of a new major antigenic variant. Synchrony is quantified as the reciprocal of the variance in onset timings for seasons, where epidemic activity for an antigenic variant was present in all five cities. *p* values are from Wilcoxon two sample tests (n = 14). Box plots show the median, first and third quartile values, as well as overall range.

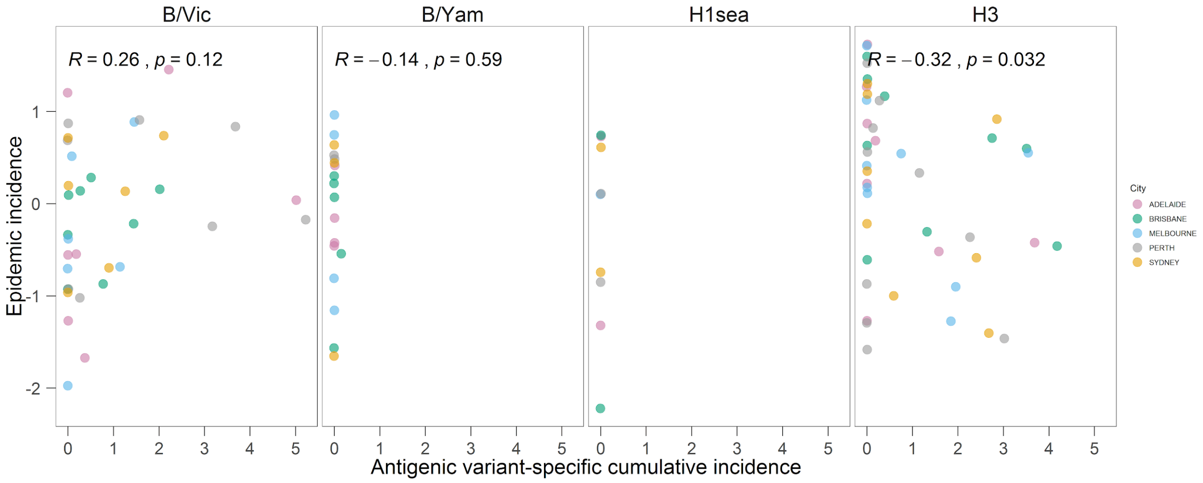

Supplementary Figure 12: Robustness of analyses of the effect of antigenic variant-specific cumulative incidence on subsequent epidemic incidence, towards potential antigenic characterisation errors. Analyses as part of main text (Figure 4) attribute all A/H3 cases in 2004 to California/7/2004 and in 2005 to A/Wisconsin/67/2005 antigenic variants, due to delays in updating vaccine strain nomenclature. Here, we make no such assumptions. Within each subtype, incidence for individual epidemics were log transformed and subtracted by the city-specific mean of log incidence, to allow for comparison between cities. Cumulative incidence was measured relative to the city-specific mean epidemic size, where 1 is equivalent to the mean epidemic incidence. *r* and *p* values are from Pearson's correlation tests (n = 37, 20, 9 and 45 for B/Vic, B/Yam, A/H1sea and A/H3 respectively). Note that antigenic variants of B/Yam and H1sea rarely initiated multiple epidemics during the study period.

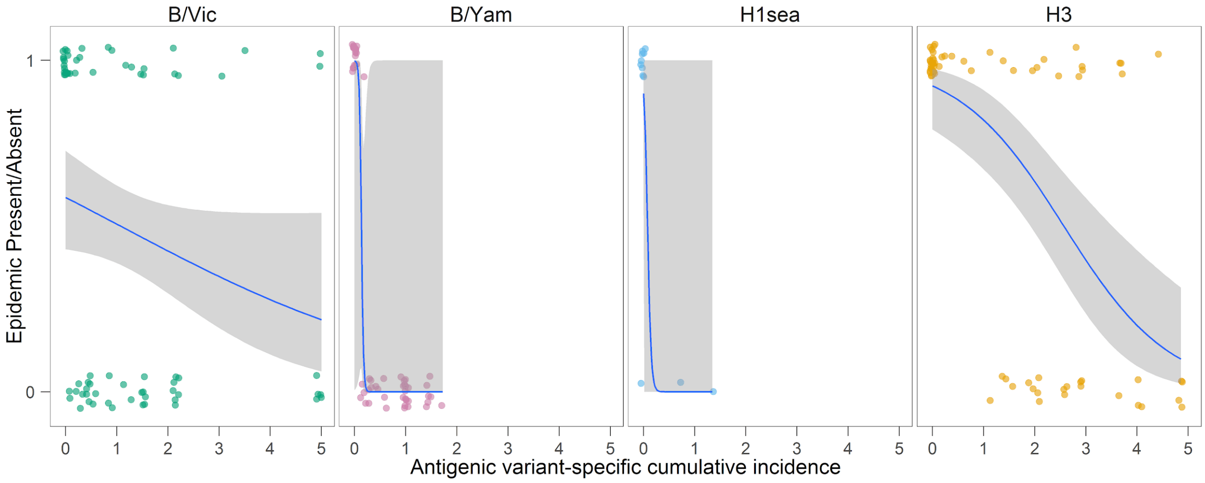

Supplementary Figure 13: Robustness of analyses of the effect of antigenic variant-specific cumulative incidence on probability of successful epidemic initiation, towards potential antigenic characterisation errors. Analyses as part of main text (Supplementary Figure 8) attribute all A/H3 cases in 2004 to California/7/2004 and in 2005 to A/Wisconsin/67/2005 antigenic variants, due to delays in updating vaccine strain nomenclature. Here, we make no such assumptions. For each antigenic variant, we examined whether epidemic levels of activity were present or absent in each of the seasons from its initial detection to its replacement by the next variant. . Antigenic variant-specific cumulative incidence was measured relative to the city-specific mean epidemic size, where 1 is equivalent to the mean epidemic incidence. Binary logistic regression models were fitted for each subtype (n = 81, 65, 13 and 72 for B/Vic, B/Yam, A/H1sea and A/H3 respectively). The 95% confidence interval is denoted by the grey shaded area. See Supplementary Table 9 for OR from the binary logistic regressions.

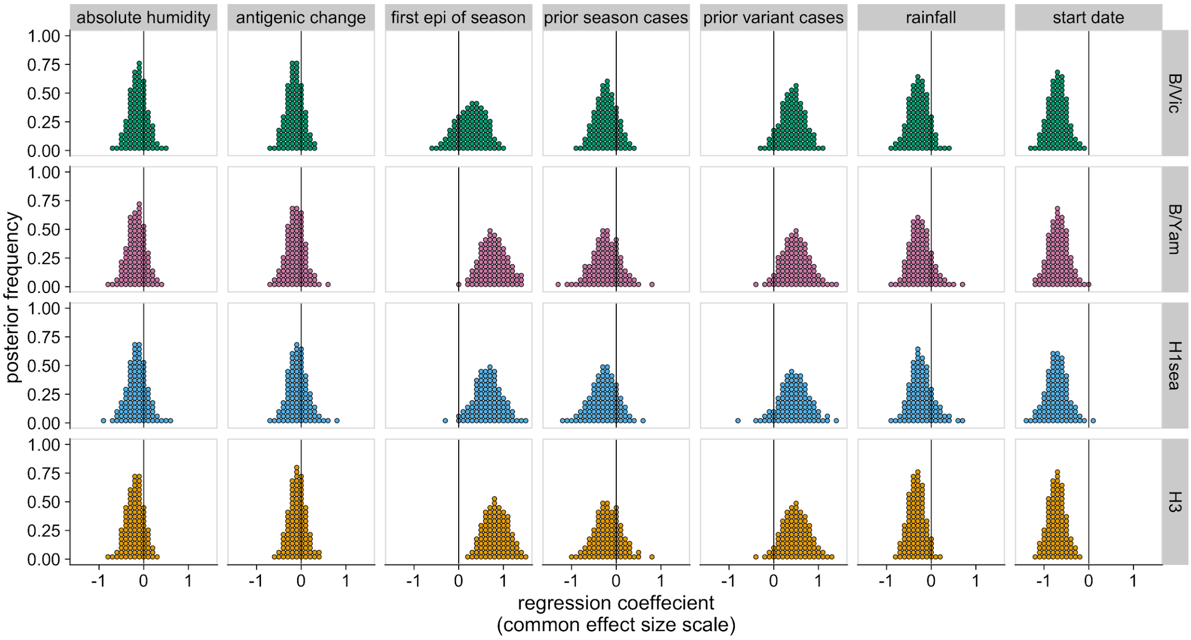

Supplementary Figure 14: Joint contributions of climatic and virological factors on epidemic incidence for individual subtypes. Using the Bayesian multilevel model, posterior distributions for the effects of climate, timing, and antigenic variables on epidemic size were estimated for individual subtypes. Both predictors and outcome variables are standardized, so effects are shown on a common scale.

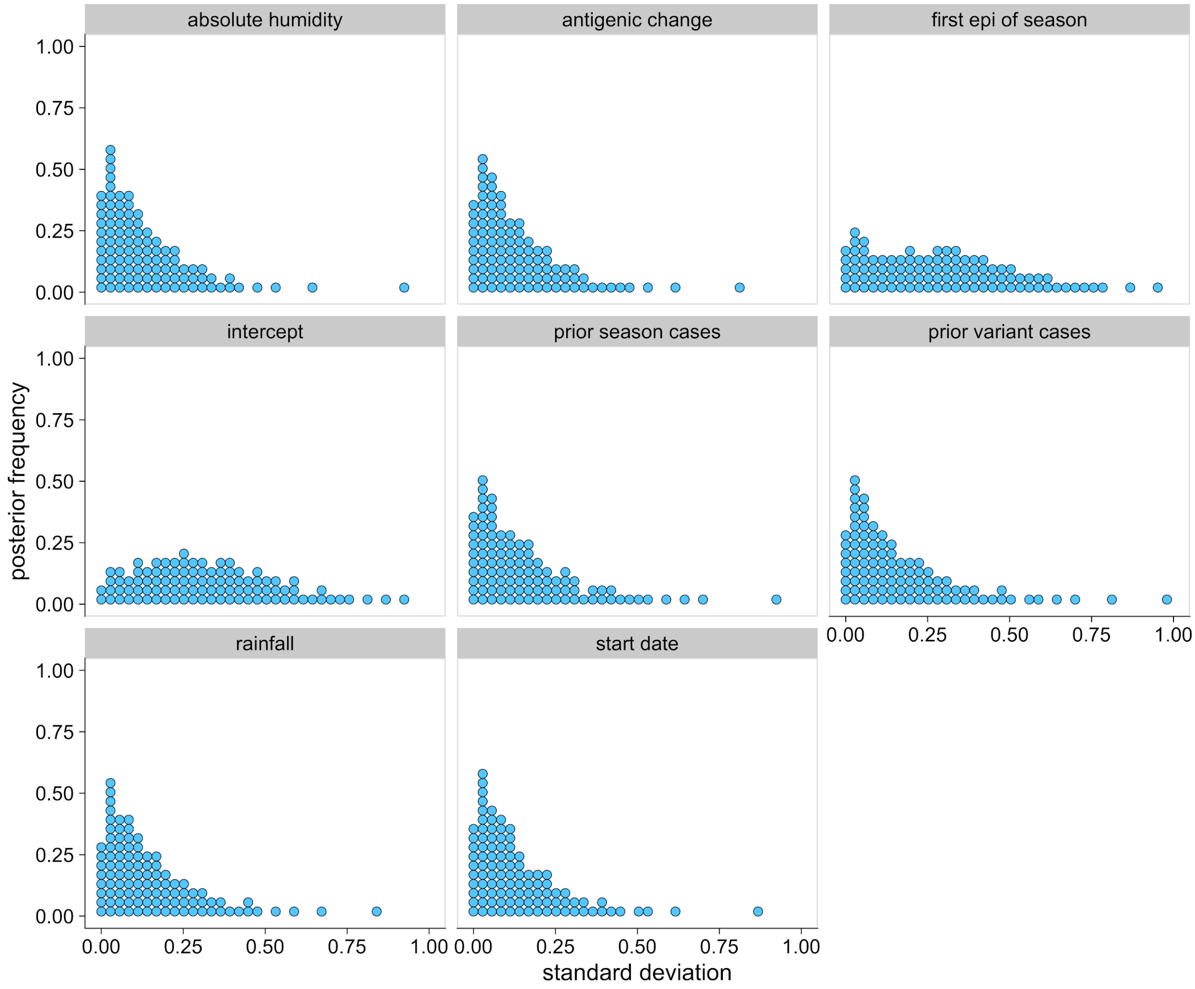

Supplementary Figure 15: Posterior distributions of standard deviations for subtype-specific effect sizes about the mean across all subtypes. Model estimates place standard deviations close to zero, suggesting that there is limited evidence from this dataset for variation among subtypes in the effects of climate, timing, or antigenicity on epidemic size.

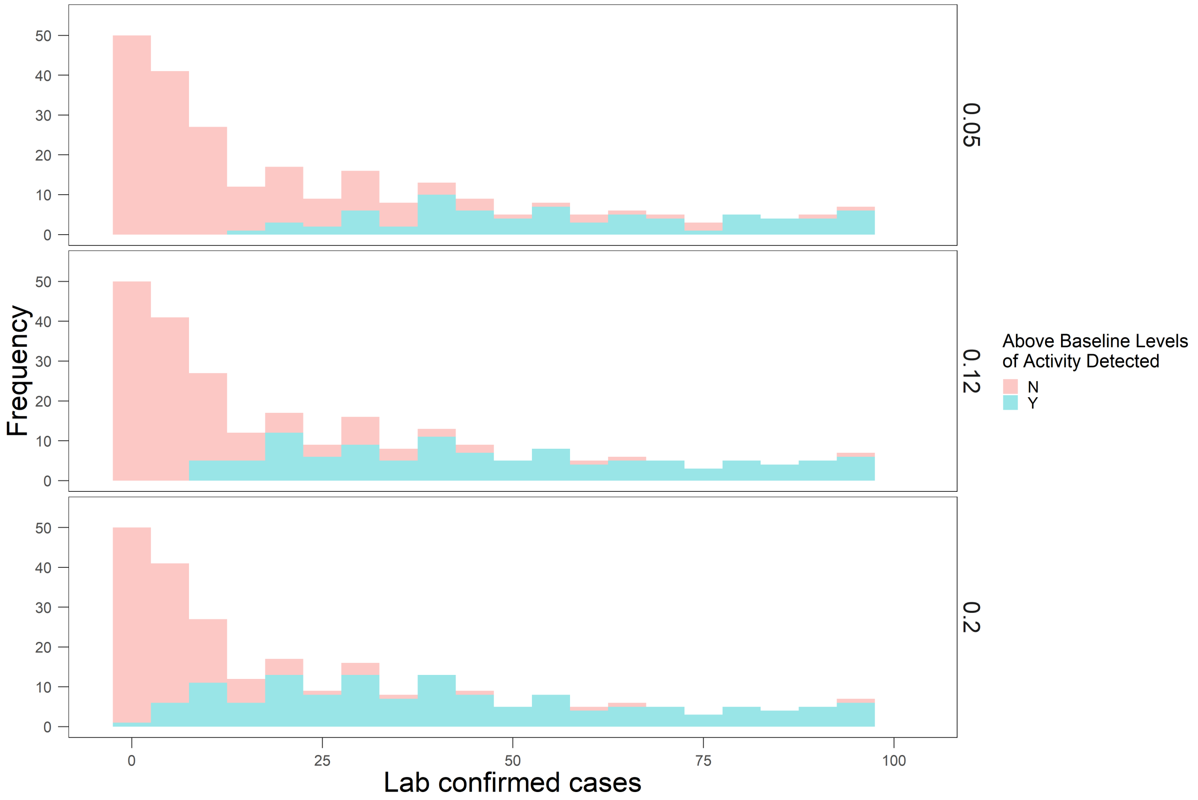

Supplementary Figure 16: Histogram of the number of lab confirmed cases for seasons with and without above baseline levels of activity. In order to focus on the sensitivity and specificity of the algorithm in identifying epidemics, for case-scarce seasons, only seasons with less than 100 cases were plotted.

The threshold value y_α, which if exceeded marks the onset of an epidemic, and thus the sensitivity of the detection algorithm are determined by the quantile parameter α. In the main text,$\alpha = 0.12$ was used, since it identified epidemic onset and end timings that corresponded well with visual inspection of the raw time series. We repeated the estimation of onset and end timings using $\alpha = 0.05$ & 0.2 (plotted in separate facets), which increased and reduced threshold values respectively. When the threshold values are lowered ($\alpha = 0.2$), there is a shift in epidemic size distribution to the left: non-epidemic activity early in the calendar year were conflated as periods of above-baseline levels of epidemic activity.

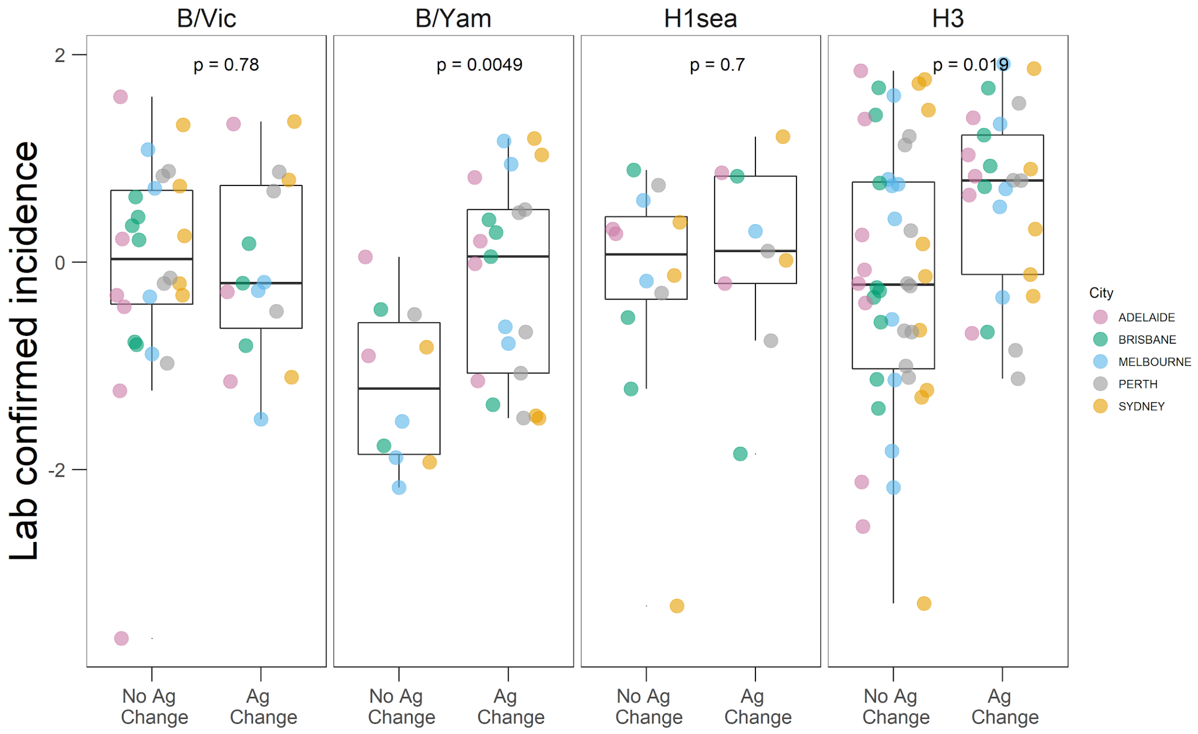

Supplementary Figure 17: Robustness of analyses of the effect of antigenic change on epidemic incidence towards lowering of epidemic detection threshold values ($\alpha = 0.2$). Reducing the threshold resulted in spurious activity being designated as epidemics, which in turn inflated the number of small epidemics observed and the apparent association between antigenic change and larger epidemics.

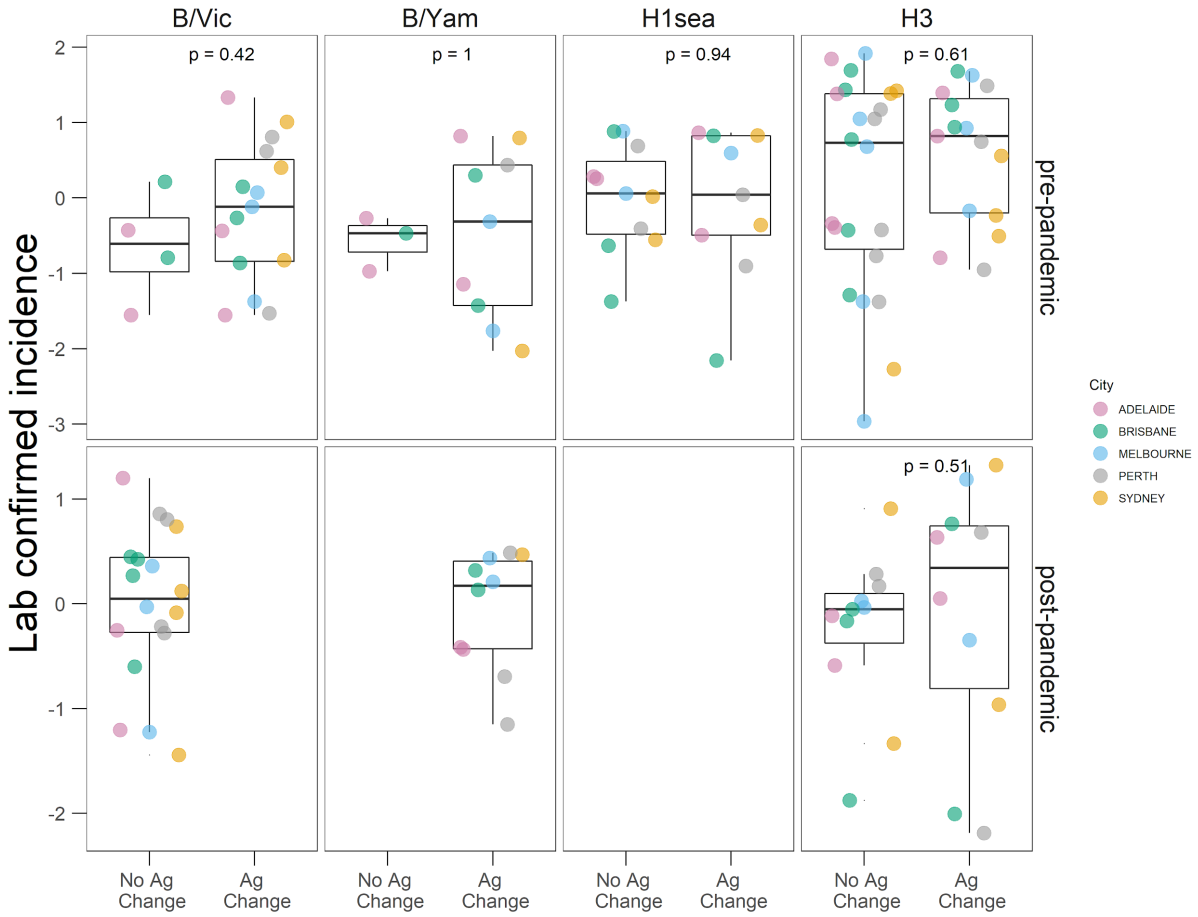

Supplementary Figure 18: Robustness of analyses of the effect of antigenic change on epidemic incidence towards potential differences in surveillance intensity between the pre- and post-pandemic eras. Within each subtype, incidence for individual epidemics were log transformed and subtracted by the city- and era-specific mean of log incidence, to allow for comparison between cities. *p* values are from Wilcoxon two sample tests. Each point corresponds to one epidemic in a city and the box plots show the median, first and third quartile of the transformed values, and range.

n-week block	Observed Mean T' (°C)	Observed Mean AH' (g m^{-3})
2	0.0294 (0.556)	-0.113 (0.304)
4	0.0158 (0.544)	-0.089 (0.304)
6	-0.0183 (0.471)	-0.0538 (0.356)

Supplementary Table 1: Observed mean climatic values over n-week continuous blocks prior to the first epidemic onset of each season. Mean T' and AH' fluctuations were aggregated across all five cities and compared against the bootstrapped distribution of random samples of observed mean T' and AH' values to determine statistical significance. The associated p values (the observed value's quantile within the bootstrap distribution) are shown in parentheses.

City	Observed Mean T' (°C)	Observed Mean AH' (g m^{-3})	n-week block
Adelaide	-0.134 (0.345)	**-0.366 (0.021)**	2
Brisbane	0.272 (0.875)	0.0967 (0.627)	2
Melbourne	-0.302 (0.154)	-0.179 (0.123)	2
Perth	0.211 (0.765)	-0.359 (0.0708)	2
Sydney	0.101 (0.675)	0.241 (0.818)	2
Adelaide	0.00971 (0.542)	-0.215 (0.0695)	4
Brisbane	0.294 (0.932)	0.18 (0.766)	4
Melbourne	-0.0572 (0.407)	-0.0117 (0.44)	4
Perth	-0.0538 (0.425)	-0.249 (0.0945)	4
Sydney	-0.113 (0.29)	-0.149 (0.236)	4
Adelaide	0.0322 (0.591)	-0.0913 (0.267)	6
Brisbane	0.0791 (0.68)	-0.0324 (0.444)	6
Melbourne	0.00498 (0.514)	-0.0201 (0.405)	6
Perth	-0.0745 (0.374)	-0.087 (0.273)	6
Sydney	-0.133 (0.217)	-0.0382 (0.406)	6

Supplementary Table 2: Observed mean climatic values over n-week continuous blocks prior to the first epidemic onset of each season. For each city, mean T' and AH' fluctuations compared against the bootstrapped distribution of random samples of observed mean T' and AH' values in order to determine statistical significance. The associated p values (the observed value's quantile within the bootstrap distribution) are shown in parentheses. Note, the result for Adelaide (bolded) were not significant after correcting for multiple testing (Holm correction).

n-week block	Observed Mean T’ (°C)	Observed Mean AH’ (g m^{-3})
2	0.0588 (0.574)	-0.219 (0.237)
4	0.0098 (0.524)	-0.235 (0.169)
6	0.0759 (0.629)	-0.133 (0.26)

Supplementary Table 3: Robustness of bootstrap analyses. Utilising only the timing estimates by Geoghegan et al.[23], we assessed if more generally, the onset of influenza A epidemic activity in the seasons from 2007 to 2015 was preceded by periods of anomalous climatic conditions; see Supplementary Discussion. Observed mean climatic values over n-week continuous blocks prior to the first epidemic onset of each season. Mean T’ and AH’ fluctuations were aggregated across all five cities and compared against the bootstrapped distribution of random samples of observed mean T’ and AH’ values, in order to calculate the statistical significance non-parametrically: the associated p values (the observed value’s quantile within the bootstrap distribution) are shown in parentheses.

City	Observed Mean T’ (^{o}C)	Observed Mean AH’ (g m^{-3})	n-week block
Adelaide	0.651 (0.948)	-0.394 (0.054)	2
Brisbane	-0.146 (0.329)	-0.208 (0.323)	2
Melbourne	-0.085 (0.425)	-0.183 (0.195)	2
Perth	-0.272 (0.267)	-0.522 (0.063)	2
Sydney	0.219 (0.754)	0.19 (0.697)	2
Adelaide	0.27 (0.826)	**-0.367 (0.029)**	4
Brisbane	-0.122 (0.328)	-0.31 (0.191)	4
Melbourne	0.176 (0.722)	-0.062 (0.352)	4
Perth	-0.328 (0.167)	-0.434 (0.052)	4
Sydney	0.085 (0.643)	-0.019 (0.468)	4
Adelaide	0.168 (0.756)	**-0.311 (0.041)**	6
Brisbane	0.01 (0.522)	-0.112 (0.353)	6
Melbourne	0.22 (0.8)	-0.027 (0.416)	6
Perth	-0.276 (0.176)	-0.299 (0.098)	6
Sydney	0.268 (0.892)	0.062 (0.601)	6

Supplementary Table 4: Robustness of bootstrap (city-level analyses). Utilising only the timing estimates by Geoghegan et al.[23], we assessed if more generally, the onset of influenza A epidemic activity in the seasons from 2007 to 2015 was preceded by periods of anomalous climatic conditions; see Supplementary Discussion. For each city, mean T’ and AH’ fluctuations compared against the bootstrapped distribution of random samples of observed mean T’ and AH’ values, in order to calculate the statistical significance non-parametrically: the associated p values (the observed value’s quantile within the bootstrap distribution) are shown in parentheses. In Adelaide, the AH’ of -0.367g m^{-3} and -0.311g m^{-3} in the 4- and 6- week blocks immediately preceding epidemics are roughly equivalent to decreases in relative humidity of 2.88% and 2.40% respectively. Both results were not statistically significant after correcting for multiple testing (Holm correction).

Subtype	Term	OR	OR adjusted SE	p value
B/Vic	(intercept)	0.946	0.276	0.848
B/Vic	Cumulative incidence	0.913	0.134	0.534
B/Yam	(intercept)	1.25	0.433	0.514
**B/Yam**	**Cumulative incidence**	**0.000213**	**0.00083**	**0.0303**
H1sea	(intercept)	3	2	0.0994
H1sea	Cumulative incidence	2.41E-06	0.00661	0.996
H3	(intercept)	3.1	1.08	0.00111
**H3**	**Cumulative incidence**	**0.705**	**0.0913**	**0.00692**

Supplementary Table 5: Binary logistic regression assessing the effect of antigenic variant-specific cumulative incidence on the probability of successful epidemic initiation for each subtype. Note: OR<1 implies that increased cumulative incidence results in a reduction in the probability of successful initiation.

Subtype	Term	OR	OR adjusted SE	p value
B/Vic	(intercept)	1	0.296	0.996
B/Vic	Cumulative incidence	0.79	0.136	0.171
B/Yam	(intercept)	0.636	0.196	0.142
**B/Yam**	**Cumulative incidence**	**2.35E-06**	**1.49E-05**	**0.0415**
H1sea	(intercept)	2	1.22	0.258
H1sea	Cumulative incidence	1.49E-06	0.00433	0.996
H3	(intercept)	1.97	0.611	0.0284
H3	Cumulative incidence	0.748	0.118	0.0663

Supplementary Table 6: Binary logistic regression assessing the effect of antigenic variant-specific cumulative incidence on the probability of successful epidemic initiation for each subtype. Analyses as part of main text (Supplementary Figure 6) attribute all A/H3 cases in 2004 to California/7/2004 and in 2005 to A/Wisconsin/67/2005 antigenic variants, due to delays in updating vaccine strain nomenclature. Here, we make no such assumptions. Note: OR<1 implies that increased cumulative incidence results in a reduction in the probability of successful initiation.

Supplementary Discussion

Robustness of inferences derived from our estimates of epidemic onset timings

Our antigenically characterised data set is relatively small: 18,250 cases. Especially in seasons with fewer cases, it can be difficult to differentiate epidemic from baseline activity. This limits the accuracy with which the timing of epidemic onset can be estimated. To check the robustness of our results to errors in estimated onset, we re-ran our analysis using estimated influenza A epidemic onset timings from a large-scale study of >450,000 Australian influenza cases[23].

Whilst the data set utilised by Geoghegan et al.[23] has many more cases than our dataset and thus might produce more accurate timing estimates, the lack of subtype level resolution means that the city-level epidemic activity recorded was the summation of underlying A/H3, A/H1sea and A/H1pdm09 virus specific activity. Nevertheless, we investigated whether or not, more generally, the onset of influenza A epidemic activity from 2007 to 2015 was preceded by periods of anomalous climatic conditions (Supplementary Tables 3-4 and Supplementary Figure 4-5). There were no biologically significant effect sizes or consistent patterns in T' or AH' prior to epidemic onset across the five cities, whether be it comparing against broader wintertime conditions (Supplementary Tables 3-4), as in Shaman et al.[10], or against long term average conditions for that particular time of the year (Supplementary Figures 4-5).

To further assess the robustness of our analyses, towards potential inaccuracies in our estimates of epidemic onset timings arising from our relatively small data set, we augmented our estimated epidemic onset timings with those of Geoghegan et al.[23] and repeated our climatic factor analyses using several different methods to impute epidemic activity within a season to a particular virus subtype. For each city and season, 1. we assumed that our timing estimate for the dominant influenza A subtype was incorrect and replaced it with the Geoghegan et al.[23] estimate; 2. we assumed that our timing estimate for the influenza A subtype that initiates epidemic activity earliest was incorrect and replaced it with the Geoghegan et al.[23] estimate; 3. in years in which the number of cases for the dominant influenza A subtype were

small or it was difficult to discern the period of epidemic from background activity, we assumed that our timing estimate was incorrect and replaced it with the Geoghegan et al.[23] estimate.

After substituting our estimates with the Geoghegan et al.[23] timings, in concordance with the above assumptions, we proceeded to identify the subtype/lineage (A/H3, A/H1sea, A/H1pdm09, B/Victoria, B/Yamagata) that initiated epidemic activity earliest within a season and re-ran our climatic analyses, comparing the climatic conditions in the two week periods preceding the earliest epidemic against average wintertime and average climatic values for that time of year (see Methods; these additional analyses can be reproduced by code included in the project Github repository). These assumed scenarios had little impact on the set of epidemic timings used for downstream analyses, due to the limited number of seasons considered by Geoghegan et al.[23]: 8 seasons from 2007-2015, since 2009 was omitted. Furthermore, discrepancies between our estimates and the Geoghegan et al. values are inconsequential whenever epidemic activity is first initiated by an influenza B virus lineage during a season.

We also repeated our analyses on the effect of antigenic change on the local timing of epidemics and temporal synchrony of epidemics between cities. Again, we substituted a subset of our estimated epidemic timings with timings from Geoghegan et al.[23], based on the same 3 sets of assumptions mentioned above. Across all replicates and set of assumptions, there was no evidence that antigenic change resulted in earlier local epidemics or more temporally synchronous epidemic activity across cities (these additional analyses can be reproduced by code included in the project Github repository).

Overall, after adjusting for multiple testing (Holm correction), the findings presented in the main text are robust against potential inaccuracies in our estimates of epidemic onset timings arising from our relatively small data set.

Sensitivity analysis of epidemic onset and end detection of algorithm

The threshold value y_α, which if exceeded marks the onset of an epidemic, and thus the sensitivity of the detection algorithm are determined by the quantile parameter α (see Eqn 4; Methods). In the main text, we chose $\alpha = 0.12$, since it identified epidemic onset and end timings that corresponded well with visual inspection of the raw time series. We repeated the estimation of onset and end timings using $\alpha = 0.05 \,\&\, 0.2$, which increased and reduced threshold values respectively. Overall, these alternative timings were similar to those originally estimated with $\alpha = 0.12$. However, timing estimates were found to be systematically earlier when utilising lower threshold values due to an increase in sensitivity. At the same time, this also reduced the specificity of the detection algorithm: spurious non-epidemic activity early in the calendar year were conflated as periods of above-baseline levels of epidemic activity and recorded as small sized epidemics on multiple occasions, (Supplementary Figure 16).

We reran our bootstrap analyses on the effects of climatic factors with these alternative epidemic timings. The estimated epidemic onset and end timings remained largely invariant to changes in α so it was unsurprising that we did not identify any fluctuations in anomalous temperature and absolute humidity in the two, four and six week periods immediately prior to the onset of the earliest epidemics from 2000-2015.

At a lower value of $\alpha = 0.05$, which increased the threshold value for detection and the specificity of the algorithm, we similarly found no evidence of consistent effects of antigenic change on epidemic size (Wilcoxon two-sample test) . In contrast, when $\alpha = 0.2$, it appeared that for B/Yam and A/H3, the epidemics were of greater size in seasons associated with the emergence of a new antigenic variant (Wilcoxon two-sample test; Supplementary Figure 17). However, this is likely to be an artefact of the reduced specificity of the detection algorithm, which inflated the number of seasons in which small so-called epidemics were detected.

We aggregated the data by week and by two-week periods and found that the latter produced smoother time series: this reduced the effect of stochastic noise and made it more amenable for

use with our detection algorithm. Aggregation by two-week periods could however obscure fluctuations in local weather, which are likely to occur at shorter timescales. Reassuringly however, we found that the detection of epidemics and estimated timings corresponded well between values calculated from data aggregated by two-week periods and by week: 239/320 instances had identical results, whilst in only 43/320 instances did timing estimates differ by more than 14 days. These relatively minor differences in timing estimates did not impact our results: we did not identify any fluctuations in anomalous temperature and absolute humidity in the two, four and six week periods (two-week aggregation) or in the one, two and three week periods (weekly aggregation) immediately prior to the onset of the earliest epidemics from 2000-2015.

Overall, our detection algorithm and downstream results from the analyses on the effects of climatic factors and antigenic change remain robust to choice of time period for the aggregation of case counts and the selection of alternative parameters, which alter the sensitivity and specificity of the algorithm (analyses can be reproduced from code included in the project Github repository).
